# Structural insights into rice KAI2 receptor provide functional implications for perception and signal transduction

**DOI:** 10.1016/j.jbc.2024.107593

**Published:** 2024-07-18

**Authors:** Angelica M. Guercio, Amelia K. Gilio, Jacob Pawlak, Nitzan Shabek

**Affiliations:** Department of Plant Biology, College of Biological Sciences, University of California - Davis, Davis, California, USA

**Keywords:** rice, karrikin, strigolactone, structure, receptor, phytohormone, alpha-beta hydrolase

## Abstract

KAI2 receptors, classified as plant α/β hydrolase enzymes, are capable of perceiving smoke-derived butenolide signals and endogenous yet unidentified KAI2-ligands (KLs). While the number of functional KAI2 receptors varies among land plant species, rice has only one KAI2 gene. Rice, a significant crop and representative of grasses, relies on KAI2-mediated Arbuscular mycorrhiza (AM) symbioses to flourish in traditionally arid and nutrient-poor environments. This study presents the first crystal structure of an active rice (*Oryza sativa*, Os*)* KAI2 hydrolase receptor. Our structural and biochemical analyses uncover grass-unique pocket residues influencing ligand sensitivity and hydrolytic activity. Through structure-guided analysis, we identify a specific residue whose mutation enables the increase or decrease of ligand perception, catalytic activity, and signal transduction. Furthermore, we investigate OsKAI2-mediated signaling by examining its ability to form a complex with its binding partner, the F-box protein DWARF3 (D3) ubiquitin ligase and subsequent degradation of the target substrate OsSMAX1, demonstrating the significant role of hydrophobic interactions in the OsKAI2-D3 interface. This study provides new insights into the diverse and pivotal roles of the OsKAI2 signaling pathway in the plant kingdom, particularly in grasses.

Rice (*Oryza sativa*) is an important crop and is often considered a representative of other grasses, which include many additional staple crops like maize (*Zea mays*), wheat (*Triticum aestivum*), and sorghum (*Sorghum bicolor*). Symbiosis with Arbuscular mycorrhiza (AM) fungi is crucial for many plants across all lineages, but its importance in grasses is especially relevant since grasses are often grown in nutrient-poor soils and AM can assist with the uptake of nitrogen and phosphate (1). Additionally, because grasses are often found in ecosystems prone to drought stress, AM symbiosis can aid in drought tolerance by improving water-use efficiency (2, 3). AM fungi also increase tolerance to biotic and abiotic factors like disease (4) and salinity (5). The more tolerant and acclimatory grasses can be, the larger yields they can produce, critical for agriculture because of their value and importance as staple crops.

In 2015, it was discovered among mounting knowledge of fungi-derived signal perception in plants (6), the receptor protein DWARF14-Like (D14L (7), often termed KAI2 (KARRIKIN INSENSITIVE 2 (8)), or HTL (HYPOSENSITIVE TO LIGHT (9, 10)) in other species, referred to as KAI2 hereafter) is necessary for rice AM colonization (11). KAI2 is part of the Karrikin (KAR)/KAI2-mediated signaling pathway which was first discovered for its ability to sense external signaling molecules from the soil called karrikins (KARs) (8). KARs are produced by the combustion of plant material, that are able to descend into the soil in post-fire rain and subsequently act as bioactive germination stimulants (12, 13). KARs were shown to stimulate germination in 1200 species of plants across 80 genera, including non-fire-relevant plants like rice (14, 15). Identification *via* reverse genetics screens in Arabidopsis and rice of the genes involved in KAR signaling revealed three currently known proteins: the receptor KAI2, an E3 ubiquitin ligase F-box type protein D3/MAX2 (DWARF3/MORE AXILLARY GROWTH2), and a transcriptional co-repressor, SMAX1/SMXL2 (SUPPRESSOR OF MAX2-1 and SMAX1-LIKE 2), that is ubiquitinated and degraded as a result of the pathway (8, 16, 17).

The KAI2-mediated signaling pathway shows homology to another phytohormone signaling pathway, strigolactone (SL), where key proteins involved in KAR signaling (KAI2, D3/MAX2, and SMAX1/2) share sequence and structural similarities with proteins in the SL signaling pathway (7, 18, 19, 20, 21, 22). SL regulates various growth and developmental processes in plants, including shoot branching, leaf growth and senescence, secondary stem thickening, formation of adventitious roots, lateral roots, root hairs, and stimulation of Striga parasitic plant germination as well as arbuscular mycorrhizal (AM) symbiosis (23). The SL signaling pathway comprises a homologous receptor to KAI2, D14 (DWARF14), the D3/MAX2 E3 ligase, and a homologous substrate SMAX1-LIKE 6, 7, and 8 (SMXL) in Arabidopsis or D53 (DWARF53) in rice (7, 18, 19, 20, 21, 22). Based on evolutionary analyses, KAR signaling genes were found to be ancestral to SL genes, indicating a role for the KAR signaling pathway pre-dating fire sensing (24, 25).

In addition to the evolutionary context of KAR and SL signaling pathways, there is mounting evidence that the KAI2-mediated signaling is triggered by yet known endogenous small molecule thusly called KL (KAI2 ligand) (26, 27, 28). This includes the striking phenotypes observed in kai2, max2, and/or smax1 mutants even in the absence of KAR. The numerous phenotypes resulting from these mutations suggest important roles for the KAI2-signaling pathway in various plant processes, including germination, seedling development, leaf shape and cuticle formation, root architecture, symbioses, and responses to abiotic and biotic stressors (8, 9, 11, 29, 30, 31, 32).

Not only is there crosstalk between the KAR and SL signaling pathways in terms of their significance in plants, but specifically, both signaling pathways influence the ability of AM to colonize plant roots. The process by which SL and AM symbioses overlap occurs first when SLs are exuded by plant roots (33). These SLs are perceived by the fungi in the soil by an unknown mechanism; in response, SLs activate fungal metabolism and hyphae branching (34, 35). However, the KAR signaling pathway plays an even greater role; the plant’s perception and symbioses with AM fungi requires the members of the KAR signaling pathway. KAI2 and D3 are necessary for AM symbiosis in rice, demonstrated by loss-of-function mutants *d14l/kai2* and *d3*, which show suppressed symbiotic activity. This is evidenced by impaired AM colonization and marker gene induction, indicating compromised function at an early presymbiotic signaling stage (11). In the KAR signaling pathway, KAI2 and D3 facilitate the proteasomal degradation of SMAX1, aligning with SMAX1's role in AM symbioses as a negative regulator. Loss of function in *smax1* leads to increased colonization compared to wildtype, along with enhanced expression of AM-response genes and elevated exudation and biosynthesis of SL (36).

Although the specific signaling molecule perceived by the KAI2 protein to initiate symbioses is unknown, by studying the receptor itself we can accelerate the search for a fungal ligand and/or the KL. The KAI2/D14 family of α/β hydrolase receptors has been studied with the aid of structural biology in several plant species. In *Striga hermonthica*, structural studies of ShHTLs have provided insights into the most sensitive of this family of receptors and their responses towards a wide variety of ligands (37). Structural studies in Arabidopsis and pea have provided insights into ligand-binding, hydrolysis dynamics, and the residues necessary for coordinating small molecules within the catalytic pocket (38, 39). Mutational analysis of pocket residues has been demonstrated to be an effective method for altering ligand sensitivity both *in vitro* and *in vivo* (38, 39, 40, 41), assisting in the investigation of receptor function and its evolution/co-evolution with KLs. In SL signaling, the D14-D3 complex has been elucidated at multiple dynamic states (42, 43). However, the structure of KAI2 in rice has not been determined or investigated, and the formation of a productive ternary complex *in vitro* involving KAI2-D3/MAX2 remains unresolved (44, 45, 46). Previous studies have highlighted several conserved residues within the D14/KAI2 family along the receptor-D3 interface, suggesting their potential contribution to this interface, yet these residues have not been thoroughly investigated (47). Furthermore, the function of the D3-KAI2 interface in perturbing the involved residues, as well as its impact on the subsequent degradation of the target of KAR-induced germination, SMAX1, has yet to be fully elucidated.

In this study, we present the first crystal structure of the KAI2 receptor in rice and use this structure to inform the mutational optimization of the receptor for synthetic ligands. Additionally, we utilize rice KAI2 as a model to further explore its interface with D3, as well as the functional implications of altering this interface on the subsequent turnover of the KAI2-pathway target, SMAX1. This study aims to offer broader insights into the mechanism by which α/β hydrolase receptor KAI2 perceives ligands, while also highlighting the significance of rice KAI2 as a representative model for other important grasses.

## Results

### The structure of rice KAI2 reveals a conserved ligand-binding pocket

**Figure 1:**
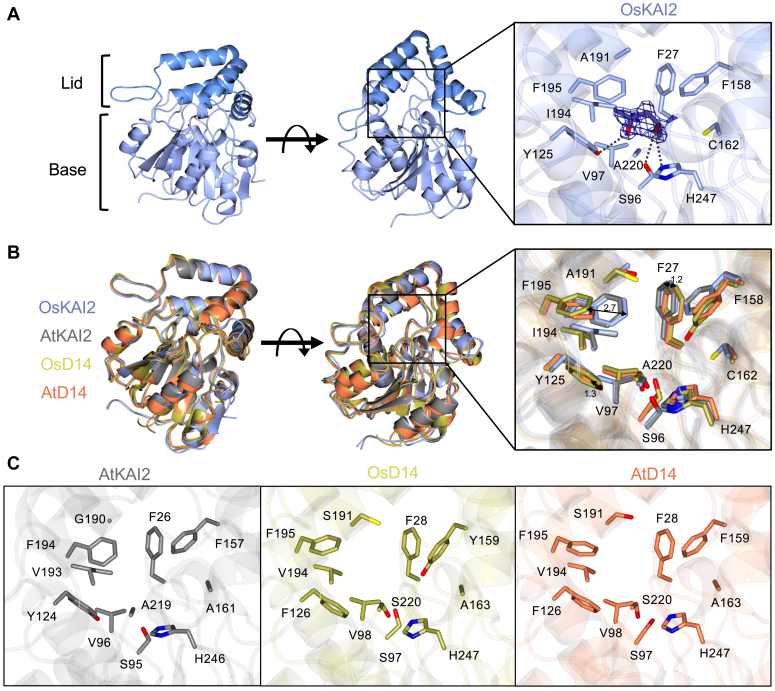
**Overview of rice KAI2 crystal structure and comparison with other relevant α/β hydrolases.***A*, overall structure of rice (Os) KAI2 with MPD bound in the active site. Lid and Base domains can be observed from the side view with lid shown in *dark blue* and base in *light blue*. The top view of the structure can be observed upon rotation. MPD is bound in the active site with all surrounding residues labeled, showing the catalytic S96 and H247 at the base of the active site and the pocket residue of interest, C162 to the *right*. The 2Fo-Fc map of the density surrounding MPD is shown to be sigma =1.0. Hydrogen bonds between MPD and neighboring residues are shown by *dashed grey lines*. *B*, superposition of OsKAI2 (*blue*) with AtKAI2 (*grey*, 4HRY), OsD14 (*gold*, 4IH9), and AtD14 (*orange*) illustrating high similarity. Both global and pocket superpositions are illustrated. *C*, isolated zoom-in from (*B*) *right*, highlighting relevant residues in AtKAI2, OsD14, and AtD14 structures. Residues are numbered according to species-specific positions.

To elucidate the mode of ligand perception, we determined the crystal structure of rice (*O. sativa*, Os) KAI2 to 1.28 Å resolution (Fig. 1*A* and Table S1). As observed in previously determined KAI2 crystal structures, OsKAI2 comprises of a canonical α/β-hydrolase fold with the binding pocket situated within the central core domain, beneath the helical lid domain (residues Tyr125-Ser197) (Figs. 1*A*, S1, and S2*A*). A Dali structural similarity search revealed that OsKAI2 has the greatest similarity to Arabidopsis (At) KAI2 with a sequence identity of 77% and RMSD of 0.5 Å over 268 Cα (Fig. S2*B*) (48).

Upon a closer inspection of the binding pocket, the conserved Ser-His-Asp catalytic triad is positioned at the base of the active site. The pocket comprises a hydrophobic cleft made up of phenylalanine, isoleucine, and valine residues, making it well-suited to bind the aromatic rings of KAR or KL-type ligands. Interestingly, we have also determined the OsKAI2 crystal structure with 2-Methyl-2,4-pentanediol (MPD, Fig. S3*A*), originating from the crystallization reagents (Table S1). The S-enantiomer of MPD is bound within the pocket, forming hydrogen bonding interactions with the key catalytic residues H247 and S96, alongside Y125 (Fig. 1*A*). Although MPD is not the natural ligand, its orientation within the pocket is likely indicative of how the endogenous ligand interacts with the pocket residues. Notably, a comparative analysis of B-factors between the apo and MPD-bound OsKAI2 showed reduced dynamics within the catalytic pocket upon MPD binding. The significant shift observed for F27 is primarily due to CH-Π contacts between MPD and F27 (Table S2). The OsKAI2 pocket was compared against that of AtKAI2 (48) which shows similar pocket residues, with the exceptions of C162 and A191 in OsKAI2, which represent smaller side-chain groups in the positions of A161 and G191 in AtKAI2 (Figs. 1, S1, and S2). The OsKAI2 and AtKAI2 structures were also compared to the analogous receptors for SL signaling in both rice OsD14 (49), and in the model plant Arabidopsis, AtD14 (Figs. 1 and S2). To that end, we determined a new AtD14 crystal structure at a higher resolution (2 Å) than the previously reported structures and was hence applied here for comparative analysis (Fig. 1 and Table S1). The AtD14 crystal structure, determined in our study, shows high similarity to the previously reported structure (PDB: 4IH4) with an RMSD of 0.557 1.2 Å over 263 Cα. Structural superposition with OsKAI2 showed an RMSD of 1.2 Å over 265 Cα and 1.2 Å over 264 Cα for OsD14 and AtD14 respectively, and both D14s gave a sequence identity of 56%, confirming high structural similarity in all four proteins examined here (Figs. 1, S1, and S2). When focusing on the binding pocket, several residues including the Ser-His-Asp catalytic triad are conserved in all proteins, highlighting its importance in the catalytic mode of action performed by α/β hydrolases. However, there are some clear differences in both the positioning and type of residue found in the pocket when comparing the KAI2s and D14s. Most noticeably, F27/F26 in OsKAI2 and AtKAI2 are shifted by 1.2 Å in respect to the positioning of F28 in both D14s (Figs. 1*B* and S1). Similarly, F195 in both KAI2s is shifted into the pocket by 2.7 Å when compared to F195 in both D14s. Furthermore, both KAI2s possess Y125/124 in place of F126 in D14s, with the hydroxyl group reaching 1.3 Å further into the pocket; similarly, both KAI2s possess the larger hydrophobic I194 in place of V194 seen in the D14s. Conversely, we found that A220 in both rice and Arabidopsis KAI2s is replaced with S220 in D14s which is slightly larger and more polar, perhaps indicating a role in strigolactone binding (Figs. 1 and S1). The OsKAI2 pocket analyses revealed a pocket measured to have a volume of 108 Å (Fig. S2) (50). This is an intermediate pocket size when compared to others in the family, such as AtKAI2 with an approximate volume of 63 Å, OsD14 at 149 Å, and ShHTL7 (51) at 215 Å (Fig. S2). Collectively, the differences between KAI2 and D14s demonstrate that distinct residues reach further into the pocket in the KAI2s, suggesting an adapted and more compact pocket to better fit KL/SL-like ligands.

### The evolutionary context of rice KAI2 indicates highly conserved grass receptors

**Figure 2:**
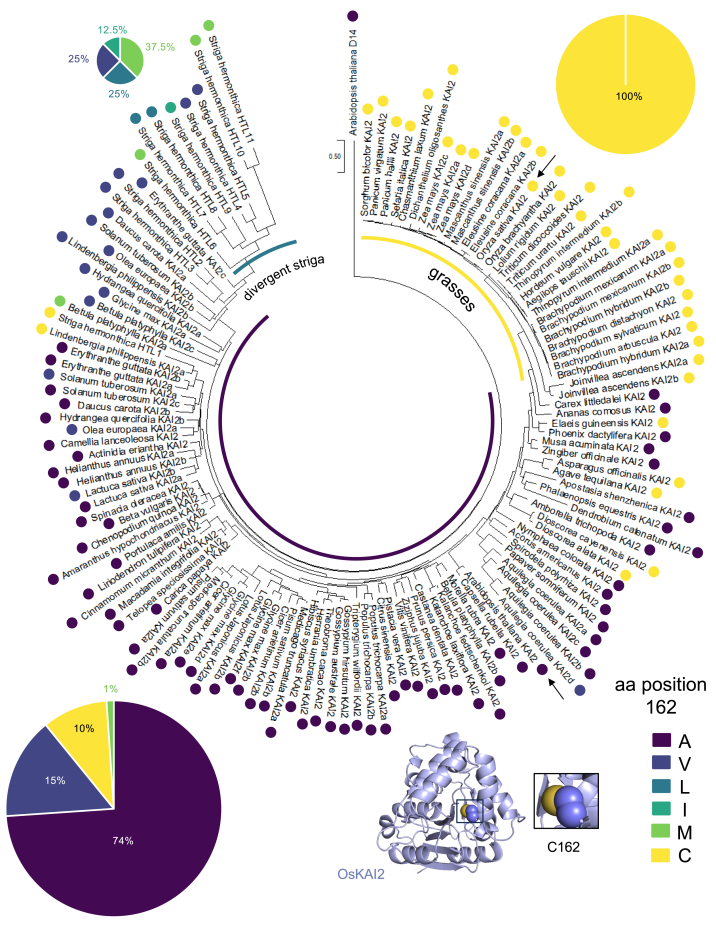
**Evolutionary context of rice KAI2 and analyses of site 162.** Maximum likelihood tree of 129 KAI2 and one D14 amino acid sequences. AtKAI2 and OsKAI2 are indicated in *tree* with *black arrows*. Colored dots on each species’s KAI2 represent the amino acid identity at the equivalent C162 position in rice. Pie charts are split to encompass three groups highlighted on trees including grasses (*top right*), striga (*top left*), and all other species (*bottom left*). Pie charts represent the proportion of species within that group with amino acids A, V, L, I, M, or C respectively at site 162. Site 162 is detailed on OsKAI2 structure (*bottom middle*). The area of the pie chart is proportional to the number of species sampled.

**Figure 3:**
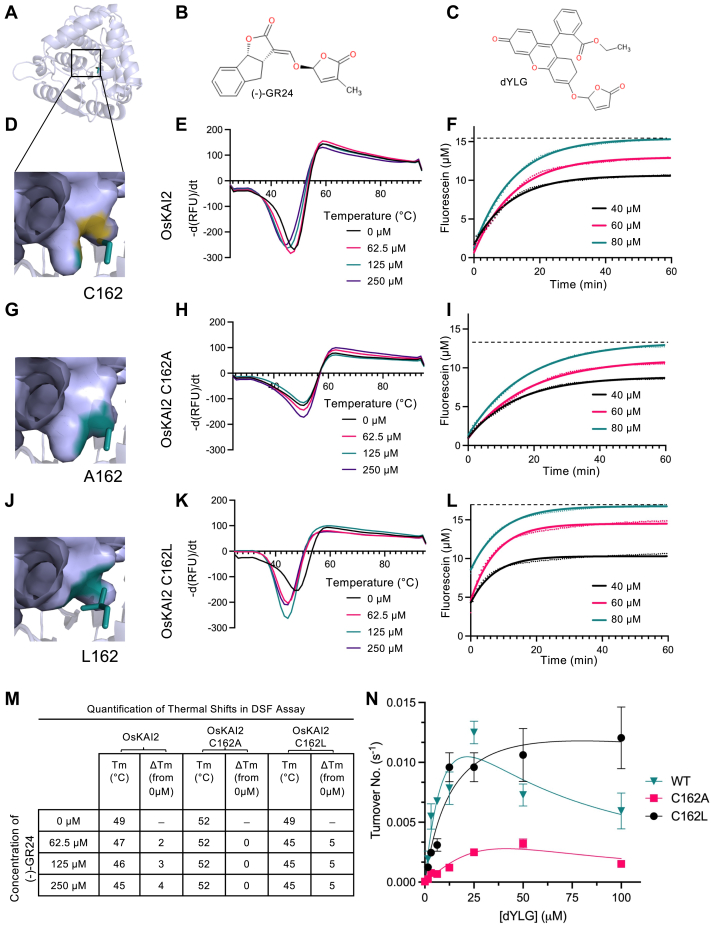
**Mutational analyses of rice KAI2 and its effect on receptor/enzymatic activity.** OsKAI2 structure is shown as a cartoon (*light blue*, *A*). Closeup views on the residue at position 162 are shown in *teal sticks* (*D*, wildtype; *G*, KAI2 C162A; and *J*, KAI2 C162L). Wildtype KAI2 (*E* and *F*), KAI2 C162A (*H* and *I*), and KAI2 C162L (*K* and *L*) were subjected to differential scanning fluorimetry in the presence of molecule (−)-GR24 (*B*), and dYLG (*C*) hydrolysis assays respectively. Quantification of melting temperature (Tm) observed in (*E*, *H*, and *K*) is provided in (*M*). In dYLG hydrolysis (*F*, *I*, and *L*) colored lines represent non-linear regression curved fit, based on average values of triplicates of raw data (shown in *dots*). *Dashed lines* represent plateau values for the highest concentration of dYLG (80 μM). *N*, dYLG hydrolysis initial rate kinetics assay.

In order to understand how KAI2 in rice has evolved within the context of the KAI2 family, a maximum likelihood tree was generated from an amino acid alignment of 131 KAI2s and one D14 (Fig. 2 and Table S3). Noticeably, based on the tree topology, KAI2s within grasses are very similar to one another. Consequently, the gene-encoded DNA sequences were examined for increased selection; using the RELAX method comparing the grass branches to the rest of the tree, the test for selection intensification (K = 30.08) was significant (*p* = 0.000, LR = 22.00) (52). As such, grasses are experiencing selective pressures, hence we employed structural studies to take a deeper look at grass-unique residues as compared to the rest of the tree. Residues within the ligand binding pocket, as illustrated in Figures 1, S1, and S2, encompass the invariant catalytic triad S96, D218, and H247. While residues F27, V97, Y125, F158, I194, F195, and A220 show some variability in other species, they exhibit minimal variations and maintain high conservation across the entire family, without specific divergence in grasses. Residue A191 has been previously reported to be a divergent residue in other species but is an alanine at this position in the majority of species assessed including in grasses. Previous studies have demonstrated how amino acid substitutions in the ligand binding pocket can influence receptor function by altering specificity and efficacy towards different ligands (23, 38, 41, 53). Strikingly, due to its distinct proximity to the ligand binding pocket, residue C162 was selected for further inspection (Figs. 1 and 2). We investigated the amino acid identities at position 162 in all species assessed within the phylogenetic tree. Each species analyzed in the phylogenetic tree is color-coded with their amino acid identity at the equivalent position 162. The pie charts plotted illustrate the proportion of each amino acid present at that position in grasses (Fig. 2, top right), the divergent family of receptors in striga (top left), and in all other representative species (Fig. 2, bottom left). Notably, upon examining all sequences, alanine emerges as the most common amino acid at position 162 (or its equivalent when aligned). This consistency is evident not only in Arabidopsis KAI2 but also in 74% of all species examined outside of grasses and striga (Figs. 2 and S1). Intriguingly, when examining some of the most active KAI2 receptors in *Striga hermonthica*, a distinct set of residues at this position has diverged, comprising leucine, valine, isoleucine, and methionine (Fig. 2). The receptor HTL7, which demonstrates heightened sensitivity to various tested ligands both *in vivo* and *in vitro*, features leucine in this position (Fig. S1). We next examined how these residues affect the activity of rice KAI2 as a receptor and enzyme by generating the substitutions C162A and C162L (Figs. 3*A* and S4).

### Perturbing the rice KAI2 binding pocket affects sensitivity towards synthetic ligands

In the absence of knowledge of the endogenous KL, synthetic ligands have proven immensely effective in better understanding the KAI2 family receptors. Specifically, KAI2 receptors have been observed to be enantio-specific (38, 54). As such, we tested the response of the wildtype (WT) OsKAI2 and the C162A and C162L mutants (Figs. 3, *A*, *D*, *G*, and *J*, and S4, *A*–*F*) towards both the (+)-GR24 and (−)-GR24 enantiomers (synthetic SL analogs, Figure 3*B* and S3). Generally, SL receptor D14 responds to (+)-GR24 due to its similarity to natural SLs, whilst KAI2 proteins tend to respond to (−)-GR24 more readily (38, 41, 54, 55, 56). Indeed, using differential scanning fluorimetry (DSF), we observed no response with any KAI2 examined to the (+)-GR24 (Fig. S4, *D*–*F*). Markedly, the WT OsKAI2 responds to increasing concentrations of (−)-GR24 as shown by a shift in the melting temperature (Tm) curve (Fig. 3*E*). Interestingly, in comparison to the WT OsKAI2, the C162A mutation seems to slightly stabilize the protein as seen by the relative rightward shift in Tm (52 °C compared to 47 °C, Fig. 3*M*); nonetheless, the mutation to alanine led to a loss of all sensitivity towards (−)-GR24 (Fig. 3*H*). This is likely due to the increased thermal stability of OsKAI2^C162A^, which compromises ligand-induced destabilization that is typically observed in these receptors. Furthermore, the C162L mutation provides an increased sensitivity towards (−)-GR24 as illustrated by at least a 2-fold increase in sensitivity (62.5 μM *vs.* 125 μM) when compared to the WT KAI2 (Fig. 3, *K* and *M*).

We next investigated the impact of the pocket mutations on receptor function by measuring enzyme activity towards pro-fluorescent synthetic ligands (Fig. S3). To that end, we employed a substrate hydrolysis-based assay using the fluorogenic derivative of Yoshimulactone Green (YLG (57), Fig. 3, *B* and *F*) which lacks a methyl group on the lactone ring (desmethyl YLG, dYLG (58)). As expected, WT OsKAI2 can effectively hydrolyze dYLG with a typical exponential to plateau curve as previously seen in this family of enzymes (Fig. 3*F*) (47). OsKAI2^C162A^ mutant is less effective at hydrolyzing the ligand than WT as evidenced by the shallower hydrolysis curves and lower plateau (Fig. 3*I*). Strikingly, the C162L mutant surpasses the wildtype in activity, reaching a plateau at a faster rate than observed with the wildtype and with a higher overall value (Fig. 3*L*). Kinetic analysis of the initial rates of hydrolysis for the two mutants and WT OsKAI2 further corroborates these observations (Figs. 3*N* and S4*G*). The characteristically low hydrolysis rates of these KAI2 enzymes resulted in observed variation between fluorescent measurements, making the determination of enzymatic constants difficult. However, the qualitative trends observed in the data are clear. We observe substrate inhibition for both the WT and C162A mutant as the enzymes’ activities drop at high ligand concentrations. However, C162L shows negligible inhibition and reaches the greatest turnover numbers, suggesting that a leucine at this position can enhance the enzyme activity. This observation has been previously hypothesized by the residue presence in a range of the most active receptors in this family.

### Insights into rice KAI2-D3 complex and its impact on SMAX1 proteasomal degradation

**Figure 4:**
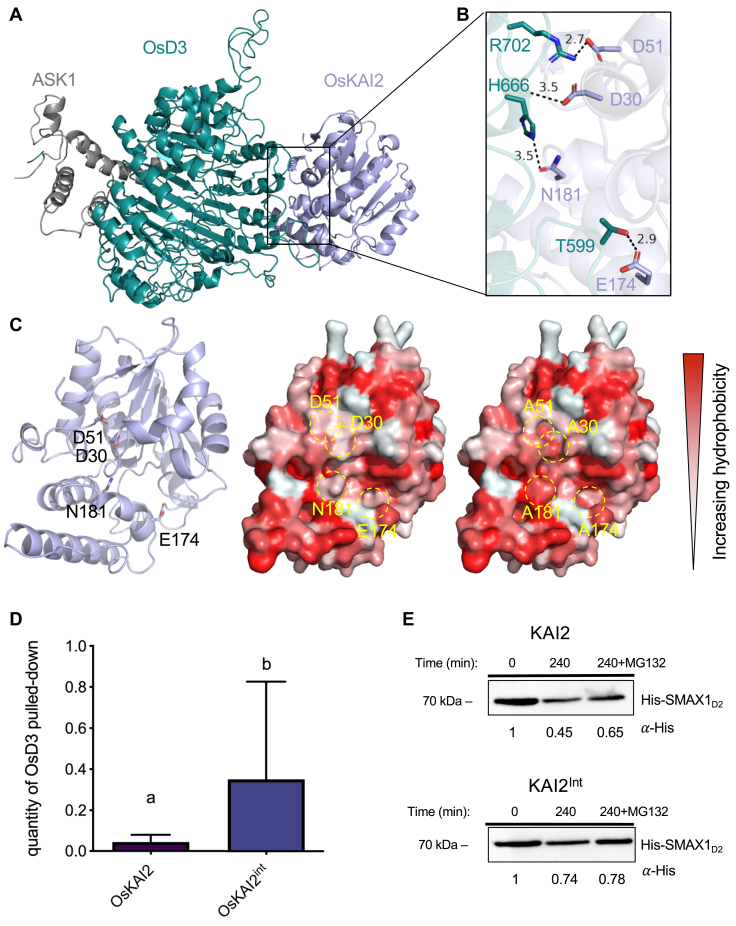
**KAI2-MAX2 interaction analysis.***A*, 3D model of OsD3-ASK1 and OsKAI2 complex (model was calculated based on PDB 5HZG using SWISS-MODEL). *B,* Zoom in view of polar residues interacting across the interface. Angstrom distances between side chains are shown in dash lines and were calculated by PyMOL. *C*, *left*: the molecular architecture of OsKAI2 determined in this study is shown in cartoon (*light blue*) and the interface residues are shown and labeled (*black*). *Middle and right*: surface representation of OsKAI2 WT structure (*middle*) and KAI2 mutant (KAI2^int^, *right*) with hydrophobicity levels shown in shades of *red*. *Dashed circles* indicate the D3-KAI2 interface polar residues that are mutated to alanine (D51A, D30A, N181A, E174A). *D*, mean values of three experimental replicas measuring quantification of pull-down of HisMSB-OsD3-ASK1 by GST-KAI2 or GST-KAI2^int^ mutant. Arbitrary units (au). (Student’s *t* test *p*  <  0.05). Proteins were resolved by SDS-PAGE and visualized *via* Western blot with ponceau and anti-His and antibodies, protein bands were quantified *in silico*. *E*, *in vitro* degradation assays using WT *Oryza sativa* cell extract monitoring HisMSB-OsSMAX1_D2_ at the indicated time points supplemented with KAI2 (*top*) or KAI2int mutant (*bottom*). Numbers under the blots indicate the amount of HisMSB-OsSMAX1_D2_ relative to the amount of HisMSB-OsSMAX1_D2_ at T  =  0). +MG132 samples indicate addition of proteasome inhibitor.

The KAR/Kl pathway relies on the ubiquitin-proteasome system to trigger transcriptional repressor degradation of SMAX1 upon ligand perception through its recruitment by KAI2 and the SKP-CULLIN1-F-BOX ubiquitin ligase SCF^D3^ (D3/MAX2 as the F-box substrate receptor). To further explore the interaction between OsKAI2 and OsD3, a 3D molecular model of the complex was generated based on the reported AtD14-D3 complex crystal structures (42, 47). The OsKAI2 sequence was threaded through the existing AtD14-D3 structure (42) wherein this model represents a unique state of the receptor undergoing conformational change revealing conserved internal residues that become exposed and accessible to form the D14/KAI2-D3 interface (47). By modeling the OsKAI2 into the complex, we identified four distinct highly conserved amino acids that are involved in KAI2/D14 and D3 interaction: D30, D51, E174, and N181 (Figs. 4, *A* and *B* and S1). These residues are within a 2.7 to 3.5 Å distance to potential interactors across the protein interface and hence participate in forming hydrogen bonds and salt bridges between the two proteins (Figs. 4*B* and S1). Subsequently, we mutated these four polar residues into small hydrophobic alanine residues to observe the effects on protein binding (OsKAI2^int^, Fig. S5, *A* and *B*). We examined the change in the binding surface’s hydrophobicity upon mutating the four interface residues to alanine and found that indeed the hydrophobicity of the surface was substantially increased (Fig. 4*C*). Interestingly, the pulldown assays in the presence of (−)-GR24 showed that the mutant OsKAI2^int^, compared to WT, exhibited increased binding to OsD3, despite the critical loss of salt bridges and hydrogen bonds (Figs. 4*D* and S5, *C* and *D*). This indicates that increasing hydrophobic interactions may enhance the receptor-E3 complex (KAI2-D3) interaction, which may not be highly favorable under physiological conditions where complex plasticity and dissociation are likely required for successful signal transduction (43, 47, 59).

To evaluate the consequences of modifying this interface on downstream signaling and the potential for plasticity as a contributing factor in the dissociation of complex components for degradation, we utilized methods previously employed (47) in *Arabidopsis thaliana*. Our aim was to examine the impact of enhancing the KAI2-D3 interaction on the proteasome-dependent degradation of the KAI2-signaling target, SMAX1. Here, we subjected SMAX1_D2_ (the D2 domain, residues 636–1041, identified in (43, 47, 59) as a sufficient degron-containing region) to a cell-free degradation assay using *O. sativa* extracts and in the presence of either recombinant KAI2 or the KAI2int mutant (Figs. 4*E* and S5, *E* and *F*). Notably, degradation of SMAX1_D2_ was slower in the presence of the KAI2int mutant (74% of proteins remaining after 240 min) compared to in the presence of wildtype KAI2 (45% of proteins remaining after 240 min). The cell extract treated with proteasome inhibitor MG132 shows the expected inhibition of SMAX1_D2_ degradation, consistent with previous observations in similar experimental settings using Arabidopsis extracts (46, 47). This finding corroborates the significance of the D3-KAI2 binding interface and emphasizes the necessity of transient interaction in the release of the complex and subsequent degradation of the substrate.

## Discussion

The endogenous roles of the KAI2-mediated signaling pathway in plants are vast and growing and currently include modulating germination, seedling establishment, hypocotyl elongation, root elongation and architecture, responses to biotic and abiotic stress, and drought (8, 9, 11, 29, 30, 31, 32). In grasses like rice, the ability to form symbiotic associations with AM fungi is paramount for nutrient uptake and stress tolerance, especially in nutrient-poor soils (1, 2, 3, 4, 5, 6). The specific physiological processes uniquely regulated by KAI2 in grasses are still being elucidated. One notable function of KAI2 receptors lies in mediating the perception of exogenous signals, volatile compounds, and those emanating as part of root signal communication with beneficial fungi (8, 11, 27, 28, 44, 60, 61). Therefore, understanding the molecular structure of KAI2 receptors offers deeper insights into how those hydrolase receptors perceive and process both exogenous and endogenous signals. In this study, we determined the first crystal structure of an active rice KAI2 hydrolase receptor, provided a comprehensive comparative analysis of this structure and its evolutionary context, and explored its biochemical function. Previous studies have examined the function of residues in the ligand-binding pocket to be adaptive, altering ligand sensitivity and/or selectivity. Within the pocket, the residue A161 in rice (position 160/161 L or M in pea and lotus) was shown to be important in differentiating ligand sensitivity between lotus and pea duplicated receptors (38, 41). Altering this residue in combination with other diverged residues led to specificity for certain enantiomers of synthetic ligand GR24 as well as implications for downstream signaling and distinct phenotypes (38, 41). Also, across all asterid KAI2s, the residue C161 (position 162 in rice) was reported as one of the four residues defining the two broad classes of KAI2s described by Martinez *et al.* (40) which are the Y124 and F124 types. The residues V96, Y124, L139, and A161 characterize the Y124-type, while residues L96, F124, I139, and V161 characterize the F124-type (40). These four sites (asterid positions 96, 124, 139, and 161) were previously tested for their function in Arabidopsis by swapping between Y124 and F124-type residues as single, double, triple, and quadruple mutants. The V161A mutation (which aligns with the C162A and C162L mutations in this study) alone was enough to significantly decrease hypocotyl elongation inhibition, but substitutions to other amino acids at this site were not examined thus far. Here, we tested alterations in position 162 in response to (−)-GR24 and dYLG ligands. Similar to observations at the phenotypic level in Arabidopsis, an alanine at this site nearly removes all sensitivity to either ligand, as determined by enzymatic and protein-level assays. This corroborates the phenotypic data in Arabidopsis at the molecular level, suggesting that this mutation results in decreased KAI2 enzyme and signaling activity (40). Interestingly, grass KAI2s contain a cysteine at this site, resulting in a receptor that is sensitive to both (−)-GR24 and dYLG ligands. However, a novel C162L substitution here improved the receptor's sensitivity to both ligands tested. A leucine in this position is shared with the most sensitive KAI2-family receptors, the HTL receptors, including HTL7 and HTL8 in *Striga hermonthica* (37). While retaining a leucine at this position seems advantageous for the perception and hydrolysis of (−)-GR24 or dYLG, there are likely several reasons why this leucine residue is not commonly observed outside of the Striga lineage naturally. As an obligate parasite, Striga is especially adapted to perceive SL-like molecules exuded from host plants (33, 37, 62, 63, 64). Striga does not germinate unless it perceives these host-exuded SLs, and it uses its highly sensitive KAI2/HTLs receptors to perceive SLs in the soil. On the other hand, grasses, as well as other species, probably utilize the KAI2 receptor for functions other than host-induced germination. The co-evolution of the KAI2 and D14 receptors and their respective small molecule perception plays a role in selecting ligand-binding pocket residues (26, 37, 38, 41, 53). Therefore, the C162 found in grasses may be important for selectively sensing grass-related KLs or grass-related KL AM signals in the soil. Taken together, our data not only corroborate previous reports on the significance of pocket residues in modulating receptor sensitivity but also reveal certain critical positions within the binding pocket of grasses' KAI2s that have evolved to sense and likely select for a highly specific yet unidentified KL.

The currently known signaling cascade following the perception of a small molecule by the KAI2 receptor involves signal transduction *via* complexing with the E3 ligase D3/MAX2 F-box protein (SCF^D3/MAX2^), which mediates the ubiquitination and subsequent degradation of the substrate, the transcriptional co-repressor SMAX1. The interface and structure of the complex formation between the receptor and the F-box protein have been of great interest (42, 43, 65). Here, we tested the function of residues predicted to localize in the KAI2-D3 interface *via* mutational analysis. Through structure-guided analysis, we have generated a KAI2-D3 model with high probability based on the D14-D3 crystal structure (42). Mutating pivotal polar residues within the KAI2-D3 interface significantly amplifies the binding between the proteins, indicating that increased hydrophobicity generates a more tightly bound and rigid complex. Thus, naturally occurring polar residues within this interface may contribute to less rigidity and weaker or transient interactions, which coincide with the characteristics of E3 ligases and their binding partners. E3 ligases are part of a timely regulated system that requires more flexible, easily dissociable interactions with their targets to allow their swift turnover by the proteasome (43, 47, 59).

Indeed, our cell-free degradation assays propose that a “tighter” receptor-E3 complex leads to a slower degradation rate of the SMAX1 target substrate. This is likely because the transient nature of the interaction has been perturbed. This data corroborates a previous study in Arabidopsis showing that conformational changes in the D3 are required to “release” the substrate for degradation by the proteasome (43, 47, 59). Future studies, conducted both in planta and at the structure-function levels, coupled with the identification of KL in grasses, will aid in elucidating the precise network of interacting residues with the E3 ligase, D3, and other binding partners, including the SMAX1 transcriptional co-repressor. Additionally, structural studies lay the groundwork for rational design strategies aimed at modulating KAI2 activity for agricultural applications, such as enhancing crop resilience and productivity (44, 45, 66, 67, 68, 69, 70).

## Experimental procedures

### KAI2 and D14 protein preparation and purification

AtKAI2, OsKAI2, the C162A mutant and the C162L mutant were independently cloned and expressed as 6× His-SUMO fusion proteins from the expression vector pAL (Addgene). BL21 (DE3) cells transformed with the expression plasmid were grown in LB broth at 16  °C to an OD600 of 0.8 and induced with 0.25 mM IPTG for 16 h. Cells were harvested, re-suspended, and lysed in extract buffer (50 mM Tris, pH 8.0, 200 mM NaCl, 5 mM imidazole, 4% Glycerol). All His-SUMO-proteins were isolated from soluble cell lysate by Ni-NTA resin. The proteins were eluted with 250 mM imidazole and subjected to anion exchange. The eluted proteins were then cleaved with TEV (tobacco etch virus) protease overnight at 4  °C. The cleaved His-SUMO tag was removed by passing through Ni-NTA resin. All proteins were concentrated by ultrafiltration to 3 to 10 mg/ml−1.

### Crystallization, data collection, and structure determination

The crystals of OsKAI2 were grown at 25  °C by the hanging-drop vapor diffusion method with 1.0 μl purified protein sample (at 12 mg/ml) mixed with an equal volume of reservoir solution containing 0.1 M Sodium HEPES, 0.1 M MOPS (acid) pH 7.5, 0.018 M Magnesium chloride hexahydrate, 0.018 M Calcium chloride dihydrate, 9.4% v/v MPD, 9.4% v/v PEG 1000, 9.4% v/v PEG 3350. The crystals of AtD14 were grown at 25  °C by the hanging-drop vapor diffusion method with 1.0 μl purified protein sample mixed with an equal volume of reservoir solution containing 0.15 M Ammonium acetate, 0.01 M Calcium chloride dihydrate, 0.1 M Tris pH 8.5, 28% broad PEG smear from BCS (Molecular Dimensions), 20% MPD. Crystals of maximum size were obtained and harvested from the reservoir solution. X-ray diffraction data was integrated and scaled with the HKL2000 package (71). OsKAI2 crystal structures were determined by molecular replacement using the AtKAI2 model (PDB: 5Z9H) (72) as the search model. AtD14 crystal structure was determined by molecular replacement using the PDB 41H4 model (49). All structural models were manually built, refined, and rebuilt with PHENIX (73) and COOT (74).

### Alignments and phylogenetic analyses

Amino acid sequences were retrieved from NCBI, Phytozome, and GenBank with accession information listed in Table S3. Multiple sequence alignment was performed in MEGA X version 10.2.6 (75) using the ClustalW algorithm (76) with a final alignment consisting of 393 positions. To identify the best-fitting model for phylogeny construction, MEGA X model testing was performed and the amino acid substitution model with the lowest BIC score (Bayesian Information Criterion) chosen for analyses was the LG+G model with 5 discrete gamma categories. The evolutionary history was inferred by using the Maximum Likelihood method and LG matrix-based model with 5 discrete gamma categories. The evolutionary history was inferred by using the Maximum Likelihood method and Le_Gascuel_2008 model (77). The tree with the highest log likelihood (−16214.51) is shown. The percentage of trees in which the associated taxa clustered together is shown next to the branches. Initial tree(s) for the heuristic search were obtained automatically by applying Neighbor-Join and BioNJ algorithms to a matrix of pairwise distances estimated using the JTT model, and then selecting the topology with superior log likelihood value. A discrete Gamma distribution was used to model evolutionary rate differences among sites (5 categories (+G, parameter = 0.6241). This analysis involved 130 amino acid sequences. There were a total of 393 positions in the final dataset. For selection analyses RELAX (52) was used in the Datamonkey (78) server with coding sequences for 100 sequences listed in Table S3. 30 sequences from evolutionary analyses were removed because of their extensive gapping. 342 sites were examined for codon dn/ds among the grass branches compared to the whole tree and showed an intensification of selection compared to the whole tree.

### Thermal shift assays

Differential Scanning Fluorimetry (DSF) experiments were performed on a CFX96 Touch Real-Time PCR Detection System (Bio-Rad Laboratories, Inc, Hercules) using excitation and emission wavelengths of 560 to 590 and 610 to 650 nm, respectively. SYPRO Orange Protein Gel Stain (Sigma Aldrich) was used as the reporter dye. Samples were heat-denatured using a linear 25 to 95  °C gradient at a rate of 1.3  °C per minute after incubation at 25  °C for 30 min in the absence of light. The denaturation curve was obtained using CFX manager software. Final reaction mixtures were prepared in triplicate in 96-well white microplates, and each reaction was carried out in 30 μl scale in reaction buffer (20 mM HEPES pH 7.3, 250 mM NaCl, 1 mM TCEP) containing 20uM protein, 0 to 250 μM ligand (as shown on the Figs. 3, *B*, *E*, and *H* and S4, *D*–*F*), 2% (v/v) DMSO, and 10× Sypro Orange (from 5000× manufacturer’s stock). Plates were incubated in darkness for 30 min before analysis. In the control reaction, DMSO was added instead of ligand. All experiments were repeated three times.

### dYLG hydrolysis

dYLG (desmethyl Yoshimulactone Green; gifted from Dr Mark Waters, UWA) hydrolysis assays were conducted in reaction buffer (50 mM MES pH 6.0, 150 mM NaCl, and 1 mM DTT) in a 50 μl volume on a 96-well, F-bottom, black plate (Greiner Bio-One). The final concentration of dimethyl sulfoxide (DMSO) was equilibrated for all samples to final concentration of 0.8%. The intensity of the fluorescence was measured by a Synergy|H1 Microplate Reader (Agilent Technologies) with excitation by 480 nm and detection by 520 nm. Readings were collected using 13-s intervals over 60 min. Background auto-YLG hydrolysis correction was performed for all samples. Raw fluorescence data were converted directly to fluorescein concentration using a standard curve. Data that were generated in Excel were transferred to GraphPad Prism 9 for graphical analysis. One-way ANOVA was performed to compare each condition with a *post hoc* Tukey multiple comparison test. All experiments were run with technical triplicates, and independent experiments were performed three times.

### Initial rates kinetic assays

dYLG activity assays were conducted in 50 μl of reaction buffer (50 mM MES pH 6.0, 250 mM NaCl, and 1 mM DTT) in a 96-well, F-bottom, black plate (Greiner), in accordance with the manufacturer's instructions. 2 uM protein was added to each well containing 0 to 80 μM dYLG and shaken for 5 s before data was collected. The intensity of the fluorescence was measured using a Synergy|H1 Microplate Reader (Agilent Technologies) with excitation at 480 nm and detection at 520 nm. Readings were collected at 2 s intervals over 2 min. The rate of YLG hydrolysis was determined from the initial linear portion of this fluorescence data in Excel and was transferred to Prism Graphpad v.10 for kinetic analysis. Kinetic constants were determined through non-linear regression based on substrate inhibition kinetics using Prism Graphpad v.10.

### Structural modeling and structure visualization

Sequence for OsKAI2 was threaded through existing AtD14–OsD3-ASK1 complex structure using SWISS-MODEL with PDB ID: 5HZG as a template. Residues of importance were identified as being within hydrogen bonding distance of potential hydrogen bond donor/acceptor pairs between OsKAI2 and OsD3. The 3D structure illustration and analysis were generated using PyMOL Molecular Graphics System, Schrödinger, LLC. Hydrophobicity was assessed using the hydrophobicity scale as defined by (79).

### Pulldown assay

OsKAI2 and the OsKAI2^int^ mutant were independently cloned and expressed as GST (Glutathione-S-Transferase) fusion protein from the expression vector pCOOL (Addgene). BL21 (DE3) cells transformed with the expression plasmid were grown in LB broth at 16 °C to an OD600 of ∼1.0 and induced with 0.2 mM IPTG for 16 h. Cells were harvested, re-suspended, and lysed in extract buffer (50 mM Tris, pH 8.5, 200 mM NaCl, 5 mM DTT, 8% glycerol). Following cell lysis, clarification, and centrifugation, the cell lysate was incubated with GST beads for 1 h at 4 °C. Beads were then washed twice with wash buffer containing 50 mM Tris, 150 mM NaCl, 1% Glycerol, and 1 mM TCEP. Beads were then washed once with 0.025% BSA blocking solution and twice with wash buffer. For control, GST beads were incubated with 0.05% BSA. Protein-bound GST beads were incubated with purified His-MSB OsD3-ASK1 (generated as in (43, 47, 59)) and 100 μM (−)-GR24 (or Acetone as control) for 30 min on ice. Following two washes with wash buffer, elution was achieved with 50 mM Tris, 10 mM Reduced glutathione pH 7.2, and 5 mM DTT. After the addition of fourfold concentrated sample buffer, boiled samples were resolved *via* SDS-PAGE, and proteins were visualized using Ponceau stain and Western blot with monoclonal anti-His (Invitrogen MA1-21315), and polyclonal anti-GST (Thermo Fisher Scientific, CAB4169) antibodies. Quantification of pulled-down OsD3 was measured by band intensity compared to the ladder 150 kDa molecular weight marker for three independent experiments. Student’s *t* test was performed to compare pull-down for wildtype compared to KAI2^int^ mutant.

### Plant cell-free degradation

Wildtype *O. sativa* seeds (Shiokari) seeds were used as a source of cell-free protein extract. Seed coat was removed and seeds were sterilized with 70% EtOH for 20 min and 30% bleach for 10 min. Germination was carried out in Petri dishes on moistened paper towels in the growth chamber at 22 °C with a 16-h: 8-h, light: dark photoperiod for 2 weeks. 150 mg of seedlings were harvested and ground in liquid nitrogen. Total proteins were extracted to a stock concentration of 8 to 10 mg ml−1 using Minute (Invent Biotechnologies Inc, SD-008/SN-009), supplemented with protease inhibitor cocktail (Roche). To monitor protein degradation in the cell-free system, 1.5 μg of purified HisMSB-OsSMAX1_D2_ protein (purified as in (47)) was incubated at 28 °C in a reaction mixture that contained, at a final volume of 12.5 μl, 20 μg of plant extract supplemented with 300 μM (−)-GR24, 40 mM Tris–HCl, pH 7.4, 4 mM ATP, 5 mM MgCl_2_, 0.8 μg/μl−1 Ub, 2 mM DTT, and 3 μg of purified OsKAI2 (wildtype) or OsKAI2^int^. Where indicated, the proteasome inhibitor MG132 was supplemented (Thermo Fisher Scientific, 47-479-01MG) as described previously (43, 47, 59, 80). Reactions were terminated at the indicated times by the addition of a fourfold concentrated sample buffer. Boiled samples were resolved *via* SDS-PAGE, and proteins were visualized using Western blot and monoclonal anti-His antibodies.

## Data availability

The atomic coordinates of structures of OsKAI2, OsKAI2 with MPD and AtKAI2 were deposited in the Protein Data Bank with accession codes 8VCZ, 8VD1, 8VD3. All relevant data are available from the corresponding author upon request.

## Supporting information

This article contains supporting information.

## Conflict of interests

The authors declare the following financial interests/personal relationships which may be considered as potential competing interests:

N. S. has an equity interest in Oerth Bio and serves on the company’s Scientific Advisory Board. The work and data submitted here have no competing interests, or other interests that might be perceived to influence the results and/or discussion reported in this paper.

## Supporting information

Guercio Supporting Information newedits

## References

[1] Smith S.E., Jakobsen I., Grønlund M., Smith F.A. (2011). Roles of arbuscular mycorrhizas in plant phosphorus nutrition: interactions between pathways of phosphorus uptake in arbuscular mycorrhizal roots have important implications for understanding and manipulating plant phosphorus acquisition. Plant Physiol..

[2] Abdalla M., Bitterlich M., Jansa J., Püschel D., Ahmed M.A. (2023). The role of arbuscular mycorrhizal symbiosis in improving plant water status under drought. J. Exp. Bot..

[3] Li J., Meng B., Chai H., Yang X., Song W., Li S. (2019). Arbuscular mycorrhizal fungi alleviate drought stress in C3 (Leymus chinensis) and C4 (Hemarthria altissima) grasses via altering antioxidant enzyme activities and photosynthesis. Front. Plant Sci..

[4] Campos-Soriano L., García-Martínez J., San Segundo B. (2012). The arbuscular mycorrhizal symbiosis promotes the systemic induction of regulatory defence-related genes in rice leaves and confers resistance to pathogen infection. Mol. Plant Pathol..

[5] Alqarawi A.A., Abd Allah E.F., Hashem A. (2014). Alleviation of salt-induced adverse impact via mycorrhizal fungi in Ephedra aphylla Forssk. J. Plant Interact..

[6] Gutjahr C., Banba M., Croset V., An K., Miyao A., An G. (2008). Arbuscular mycorrhiza-specific signaling in rice transcends the common symbiosis signaling pathway. Plant Cell.

[7] Arite T., Umehara M., Ishikawa S., Hanada A., Maekawa M., Yamaguchi S. (2009). D14, a strigolactone-Insensitive mutant of rice, shows an accelerated outgrowth of tillers. Plant Cell Physiol..

[8] Waters M.T., Nelson D.C., Scaffidi A., Flematti G.R., Sun Y.K., Dixon K.W. (2012). Specialisation within the DWARF14 protein family confers distinct responses to karrikins and strigolactones in Arabidopsis. Development.

[9] Sun X.D., Ni M. (2011). HYPOSENSITIVE to LIGHT, an alpha/beta fold protein, acts downstream of ELONGATED HYPOCOTYL 5 to regulate seedling de-etiolation. Mol. Plant.

[10] Xu Y., Miyakawa T., Nosaki S., Nakamura A., Lyu Y., Nakamura H. (2018). Structural analysis of HTL and D14 proteins reveals the basis for ligand selectivity in Striga. Nat. Commun..

[11] Gutjahr C., Gobbato E., Choi J., Riemann M., Johnston M.G., Summers W. (2015). Rice perception of symbiotic arbuscular mycorrhizal fungi requires the karrikin receptor complex. Science.

[12] Flematti G.R., Ghisalberti E.L., Dixon K.W., Trengove R.D. (2004). A compound from smoke that promotes seed germination. Science.

[13] Van Staden J., Jäger A.K., Light M.E., Burger B.V. (2004). Isolation of the major germination cue from plant-derived smoke. South Afr. J. Bot..

[14] Dixon K.W., Merritt D.J., Flematti G.R., Ghisalberti E.L. (2009). Karrikinolide - a phytoreactive compound derived from smoke with applications in horticulture, ecological restoration and agriculture. Acta Horticulturae.

[15] Kulkarni M., Sparg S., Light M., Van Staden J. (2006). Stimulation of rice (Oryza sativa L.) seedling vigour by smoke-water and butenolide. J. Agron. Crop Sci..

[16] Nelson D.C., Scaffidi A., Dun E.A., Waters M.T., Flematti G.R., Dixon K.W. (2011). F-box protein MAX2 has dual roles in karrikin and strigolactone signaling in Arabidopsis thaliana. Proc. Natl. Acad. Sci. U. S. A..

[17] Stanga J.P., Smith S.M., Briggs W.R., Nelson D.C. (2013). SUPPRESSOR of more AXILLARY GROWTH2 1 controls seed germination and seedling development in arabidopsis. Plant Physiol..

[18] Jiang L., Liu X., Xiong G., Liu H., Chen F., Wang L. (2013). DWARF 53 acts as a repressor of strigolactone signalling in rice. Nature.

[19] Soundappan I., Bennett T., Morffy N., Liang Y., Stanga J.P., Abbas A. (2015). SMAX1-LIKE/D53 family members enable distinct MAX2-dependent responses to strigolactones and karrikins in arabidopsis the plant. Cell.

[20] Zhou F., Lin Q., Zhu L., Ren Y., Zhou K., Shabek N. (2013). D14-SCF D3 -dependent degradation of D53 regulates strigolactone signalling. Nature.

[21] Gao Z., Qian Q., Liu X., Yan M., Feng Q., Dong G. (2009). Dwarf 88, a novel putative esterase gene affecting architecture of rice plant. Plant Mol. Biol..

[22] Liu W., Wu C., Fu Y., Hu G., Si H., Zhu L. (2009). Identification and characterization of HTD2: a novel gene negatively regulating tiller bud outgrowth in rice. Planta.

[23] Guercio A.M., Palayam M., Shabek N. (2023). Strigolactones: diversity, perception, and hydrolysis. Phytochem. Rev..

[24] Bythell-Douglas R., Rothfels C.J., Stevenson D.W.D., Graham S.W., Wong G.K.S., Nelson D.C. (2017). Evolution of strigolactone receptors by gradual neo-functionalization of KAI2 paralogues. BMC Biol..

[25] Delaux P.M., Xie X., Timme R.E., Puech-Pages V., Dunand C., Lecompte E. (2012). Origin of strigolactones in the green lineage. New Phytol..

[26] Conn C.E., Bythell-Douglas R., Neumann D., Yoshida S., Whittington B., Westwood J.H. (2015). Convergent evolution of strigolactone perception enabled host detection in parasitic plants. Science.

[27] Conn C.E., Nelson D.C. (2016). Evidence that KARRIKIN-INSENSITIVE2 (KAI2) receptors may perceive an unknown signal that is not karrikin or strigolactone. Front. Plant Sci..

[28] Stirling S.A., Guercio A.M., Patrick R.M., Huang X.Q., Bergman M.E., Dwivedi V. (2024). Volatile communication in plants relies on a KAI2-mediated signaling pathway. Science.

[29] Nelson D.C., Flematti G.R., Riseborough J.A., Ghisalberti E.L., Dixon K.W., Smitha S.M. (2010). Karrikins enhance light responses during germination and seedling development in Arabidopsis thaliana. Proc. Natl. Acad. Sci. U. S. A..

[30] Li W., Nguyen K.H., Chu H.D., Ha C.V., Watanabe Y., Osakabe Y. (2017). The karrikin receptor KAI2 promotes drought resistance in Arabidopsis thaliana. PLoS Genet..

[31] Wang L., Waters M.T., Smith S.M. (2018). Karrikin-KAI2 signalling provides Arabidopsis seeds with tolerance to abiotic stress and inhibits germination under conditions unfavourable to seedling establishment. New Phytol..

[32] Meng Y., Varshney K., Incze N., Badics E., Kamran M., Davies S.F. (2022). KARRIKIN INSENSITIVE2 regulates leaf development, root system architecture and arbuscular-mycorrhizal symbiosis in Brachypodium distachyon. Plant J..

[33] Cook C.E., Whichard L.P., Turner B., Wall M.E., Egley G.H. (1966). Germination of witchweed (striga lutea lour.): isolation and properties of a potent stimulant. Science.

[34] Besserer A., Puech-Pagès V., Kiefer P., Gomez-Roldan V., Jauneau A., Roy S. (2006). Strigolactones stimulate arbuscular mycorrhizal fungi by activating mitochondria. PLoS Biol..

[35] Akiyama K., Matsuzaki K.I., Hayashi H. (2005). Plant sesquiterpenes induce hyphal branching in arbuscular mycorrhizal fungi. Nature.

[36] Choi J., Lee T., Cho J., Servante E.K., Pucker B., Summers W. (2020). The negative regulator SMAX1 controls mycorrhizal symbiosis and strigolactone biosynthesis in rice. Nat. Commun..

[37] Toh S., Holbrook-Smith D., Stogios P.J., Onopriyenko O., Lumba S., Tsuchiya Y. (2015). Structure-function analysis identifies highly sensitive strigolactone receptors in Striga. Science.

[38] Guercio A.M., Torabi S., Cornu D., Dalmais M., Bendahmane A., Le Signor C. (2022). Structural and functional analyses explain Pea KAI2 receptor diversity and reveal stereoselective catalysis during signal Perception. Commun. Biol..

[39] Yao J., Mashiguchi K., Scaffidi A., Akatsu T., Melville K.T., Morita R. (2018). An allelic series at the KARRIKIN INSENSITIVE 2 locus of Arabidopsis thaliana decouples ligand hydrolysis and receptor degradation from downstream signalling. Plant J..

[40] Martinez S.E., Conn C.E., Guercio A.M., Sepulveda C., Fiscus C.J., Koenig D. (2022). A KARRIKIN INSENSITIVE2 paralog in lettuce mediates highly sensitive germination responses to karrikinolide. Plant Physiol..

[41] Carbonnel S., Torabi S., Griesmann M., Bleek E., Tang Y., Buchka S. (2020). Lotus japonicus karrikin receptors display divergent ligand-binding specificities and organ-dependent redundancy. PLoS Genet..

[42] Yao R., Ming Z., Yan L., Li S., Wang F., Ma S. (2016). DWARF14 is a non-canonical hormone receptor for strigolactone. Nature.

[43] Shabek N., Ticchiarelli F., Mao H., Hinds T.R., Leyser O., Zheng N. (2018). Structural plasticity of D3–D14 ubiquitin ligase in strigolactone signalling. Nature.

[44] Toh S., Holbrook-Smith D., Stokes M.E., Tsuchiya Y., McCourt P. (2014). Detection of parasitic plant suicide germination compounds using a high-throughput Arabidopsis HTL/KAI2 strigolactone perception system. Chem. Biol..

[45] Holbrook-Smith D., Toh S., Tsuchiya Y., McCourt P. (2016). Small-molecule antagonists of germination of the parasitic plant Striga hermonthica. Nat. Chem. Biol..

[46] Khosla A., Morffy N., Li Q., Faure L., Chang S.H., Yao J. (2020). Structure–Function analysis of SMAX1 reveals domains that mediate its karrikin-induced proteolysis and interaction with the receptor KAI2. Plant Cell.

[47] Tal L., Guercio A.M., Varshney K., Young A., Gutjahr C., Shabek N. (2023). C-terminal conformational changes in SCF-D3/MAX2 ubiquitin ligase are required for KAI2-mediated signaling. New Phytol..

[48] Bythell-Douglas R., Waters M.T., Scaffidi A., Flematti G.R., Smith S.M., Bond C.S. (2013). The structure of the karrikin-insensitive protein (KAI2) in Arabidopsis thaliana. PLoS One.

[49] Zhao L.H., Edward Zhou X., Wu Z.S., Yi W., Xu Y., Li S. (2013). Crystal structures of two phytohormone signal-transducing α/β hydrolases: karrikin-signaling KAI2 and strigolactone-signaling DWARF14. Cell Res..

[50] Tian W., Chen C., Lei X., Zhao J., Liang J. (2018). CASTp 3.0: computed atlas of surface topography of proteins. Nucleic Acids Res..

[51] Shahul Hameed U., Haider I., Jamil M., Guo X., Zarban R.A., Kim D. (2022). Structural basis for specific inhibition of the highly sensitive ShHTL7 receptor. EMBO Rep..

[52] Wertheim J.O., Murrell B., Smith M.D., Kosakovsky Pond S.L., Scheffler K. (2014). RELAX: detecting relaxed selection in a phylogenetic framework. Mol. Biol. Evol..

[53] Arellano-Saab A., Bunsick M., Galib H.A., Zhao W., Schuetz S., Bradley J.M. (2021). Three mutations repurpose a plant karrikin receptor to a strigolactone receptor. Proc. Natl. Acad. Sci. U. S. A..

[54] de Saint Germain A., Jacobs A., Brun G., Pouvreau J.B., Braem L., Cornu D. (2021). A Phelipanche ramosa KAI2 protein perceives strigolactones and isothiocyanates enzymatically. Plant Commun..

[55] Scaffidi A., Waters M.T., Sun Y.K., Skelton B.W., Dixon K.W., Ghisalberti E.L. (2014). Strigolactone hormones and their stereoisomers signal through two related receptor proteins to induce different physiological responses in arabidopsis. Plant Physiol..

[56] de Saint Germain A., Clavé G., Badet-Denisot M.A., Pillot J.P., Cornu D., Le Caer J.P. (2016). An histidine covalent receptor and butenolide complex mediates strigolactone perception. Nat. Chem. Biol..

[57] Tsuchiya Y., Yoshimura M., Sato Y., Kuwata K., Toh S., Holbrook-Smith D. (2015). Probing strigolactone receptors in Striga hermonthica fluorescence. Science.

[58] Yao J., Scaffidi A., Meng Y., Melville K.T., Komatsu A., Khosla A. (2021). Desmethyl butenolides are optimal ligands for karrikin receptor proteins. New Phytol..

[59] Tal L., Palayam M., Ron M., Young A., Britt A., Shabek N. (2022). A conformational switch in the SCF-D3/MAX2 ubiquitin ligase facilitates strigolactone signalling. Nat. Plants.

[60] Nelson D.C., Flematti G.R., Ghisalberti E.L., Dixon K.W., Smith S.M. (2012). Regulation of seed germination and seedling growth by chemical signals from burning vegetation. Annu. Rev. Plant Biol..

[61] Guo Y., Zheng Z., La Clair J.J., Chory J., Noel J.P. (2013). Smoke-derived karrikin perception by the a/B hydrolase KAI2 from Arabidopsis. Proc. Natl. Acad. Sci. U. S. A..

[62] Mangnus E.M., Zwanenburg B. (1992). Tentative molecular mechanism for germination stimulation of striga and orobanche seeds by strigol and its synthetic analogues. J. Agric. Food Chem..

[63] Zwanenburg B., Mwakaboko A.S., Reizelman A., Anilkumar G., Sethumadhavan D. (2009). Structure and function of natural and synthetic signallingmolecules in parasitic weed germination. Pest Manag. Sci..

[64] Yoneyama K., Awad A.A., Xie X., Yoneyama K., Takeuchi Y. (2010). Strigolactones as germination stimulants for root parasitic plants. Plant Cell Physiol..

[65] Seto Y., Yasui R., Kameoka H., Tamiru M., Cao M., Terauchi R. (2019). Strigolactone perception and deactivation by a hydrolase receptor DWARF14. Nat. Commun..

[66] Arellano-Saab A., McErlean C.S.P., Lumba S., Savchenko A., Stogios P.J., McCourt P. (2022). A novel strigolactone receptor antagonist provides insights into the structural inhibition, conditioning, and germination of the crop parasite Striga. J. Biol. Chem..

[67] Boyer F.D., Germain A.d.S., Pillot J.P., Pouvreau J.B., Chen V.X., Ramos S. (2012). Structure-activity relationship studies of strigolactone-related molecules for branching inhibition in garden pea: molecule design for shoot branching. Plant Physiol..

[68] Mwakaboko A.S., Zwanenburg B. (2011). Strigolactone analogs derived from ketones using a working model for germination stimulants as a blueprint. Plant Cell Physiol..

[69] Nefkens G.H.L., Thuring J.W.J.F., Beenakkers M.F.M., Zwanenburg B. (1997). Synthesis of a phthaloylglycine-derived strigol analogue and its germination stimulatory activity toward seeds of the parasitic weeds striga hermonthica and orobanche crenata. J. Agric. Food Chem..

[70] Wang D.W., Yu S.Y., Pang Z.L., Ma D.J., Liang L., Wang X. (2021). Discovery of a broad-spectrum fluorogenic agonist for strigolactone receptors through a computational approach. J. Agric. Food Chem..

[71] Otwinowski Z., Minor W. (1997). Processing of X-ray diffraction data collected in oscillation mode. Methods Enzymol..

[72] Lee I., Kim K., Lee S., Lee S., Hwang E., Shin K. (2018). A missense allele of KARRIKIN-INSENSITIVE2 impairs ligand-binding and downstream signaling in Arabidopsis thaliana. J. Exp. Bot..

[73] Adams P.D., Afonine P.V., Bunkóczi G., Chen V.B., Davis I.W., Echols N. (2010). PHENIX: a comprehensive Python-based system for macromolecular structure solution. Acta Crystallogr. Sect. D: Biol. Crystallogr..

[74] Emsley P., Lohkamp B., Scott W.G., Cowtan K. (2010). Features and development of coot. Acta Crystallogr. Sect. D: Biol. Crystallogr..

[75] Kumar S., Stecher G., Li M., Knyaz C., Tamura K. (2018). Mega X: molecular evolutionary genetics analysis across computing platforms. Mol. Biol. Evol..

[76] Larkin M.A., Blackshields G., Brown N.P., Chenna R., McGettigan P.A., McWilliam H. (2007). Clustal W and clustal X version 2.0. Bioinformatics.

[77] Le S.Q., Gascuel O. (2008). An improved general amino acid replacement matrix. Mol. Biol. Evol..

[78] Weaver S., Shank S.D., Spielman S.J., Li M., Muse S.V., Kosakovsky Pond S.L. (2018). Datamonkey 2.0: a modern web application for characterizing selective and other evolutionary process. Mol. Biol. Evol..

[79] Eisenberg D., Schwarz E., Komaromy M., Wall R. (1984). Analysis of membrane and surface protein sequences with the hydrophobic moment plot. J. Mol. Biol..

[80] Ganapathy J., Hand KA., Shabek N. (2024). Analysis of 26S proteasome activity across Arabidopsis tissues. Plants.

