# Supplementary material for: Structural insights into rice KAI2 receptor provide functional implications for perception and signal transduction
Source: J Biol Chem. 2024 Jul 18;300(8):107593. doi: 10.1016/j.jbc.2024.107593 (PMC11350264; doi:10.1016/j.jbc.2024.107593)
Supplement: Guercio Supporting Information newedits [file mmc1.pdf]

Supporting Information

Structural insights into rice KAI2 receptor provide functional implications for perception and signal transduction

Angelica M. Guercio, Amelia K. Gilio, Jacob Pawlak, and Nitzan Shabek*

Figure S1. Multiple sequence alignment of key KAI2/D14 sequences

Figure S2. Comparing overall structure and pocket residues of OsKAI2 with other KAI2/D14 proteins

Figure S3. Structures of molecules discussed in this manuscript

Figure S4. KAI2s used in this study and additional biochemical data

Figure S5. Reagents and gels corresponding to pull-down and in vitro degradation assays.

Table S1. Data collection, phasing and refinement statistics

Table S2. Comparison of B-factors for selected residues within the catalytic pocket.

Table S3. Sequences used in amino acid tree and dN/dS analysis.

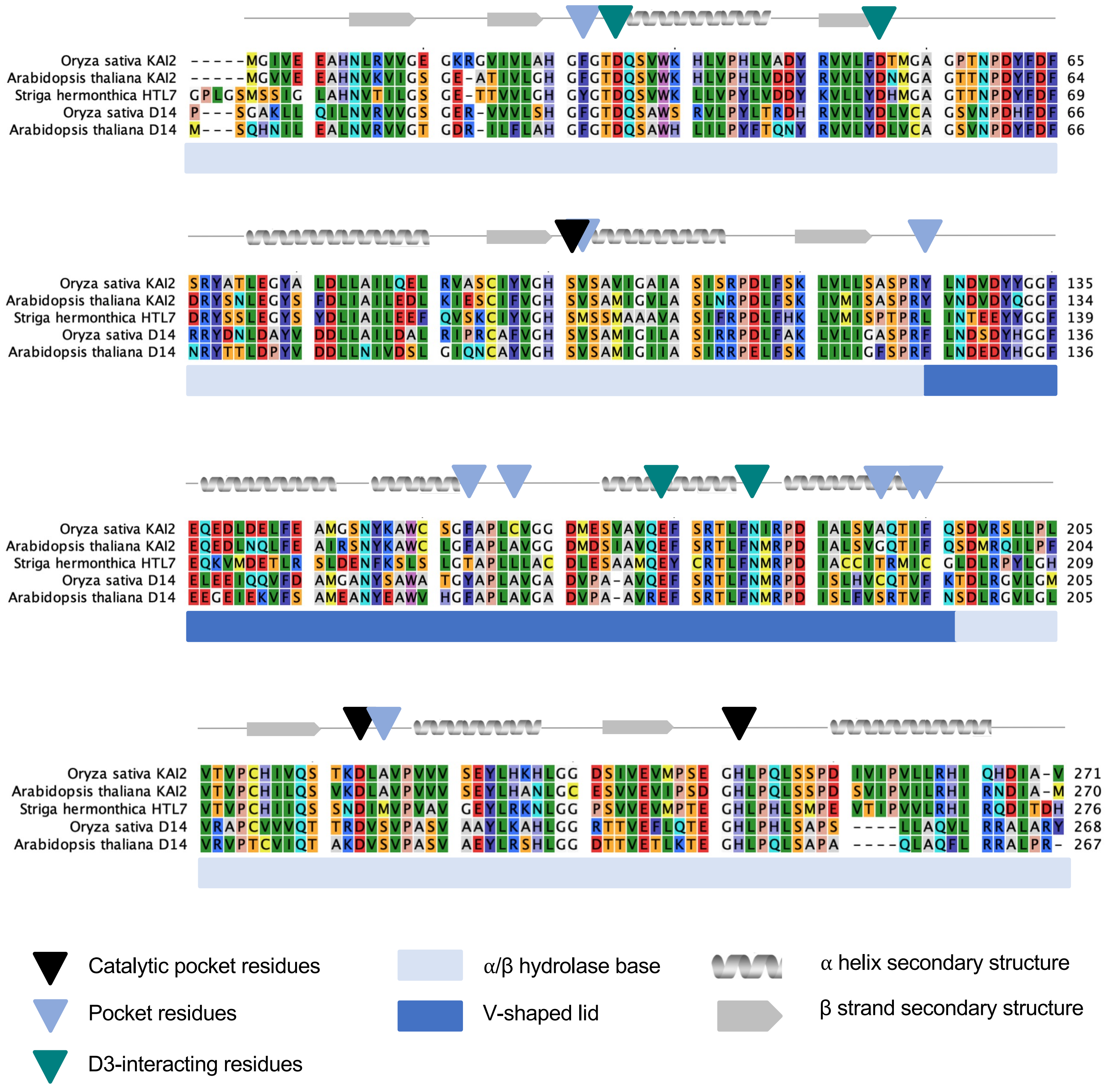

Figure S1. Multiple sequence alignment of key KAI2/D14 sequences. Amino acid alignment of four KAI2/D14s. Residues are colored in Rasmol standard colors. Numbers above residues refer to position in alignment. Black arrows refer to catalytic pocket residues S96, D218, and H247. Light blue arrows point out residues in pocket featured in main Fig. 1. Dark teal arrows highlight residues mutated in OsKAI2^{int} mutant in Fig. 4. OsKAI2 lid (light blue) and base (dark blue) domains are indicated below alignment. Secondary structure of OsKAI2 is shown above sequence in gray helices, beta strands, and non-secondary structure containing loops as lines.

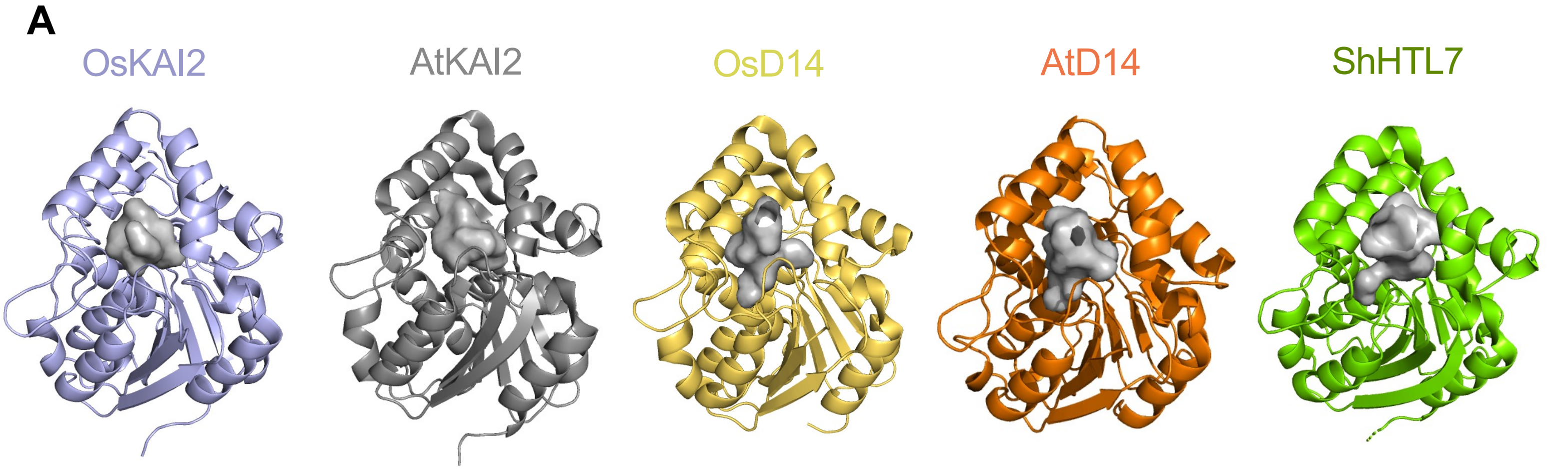

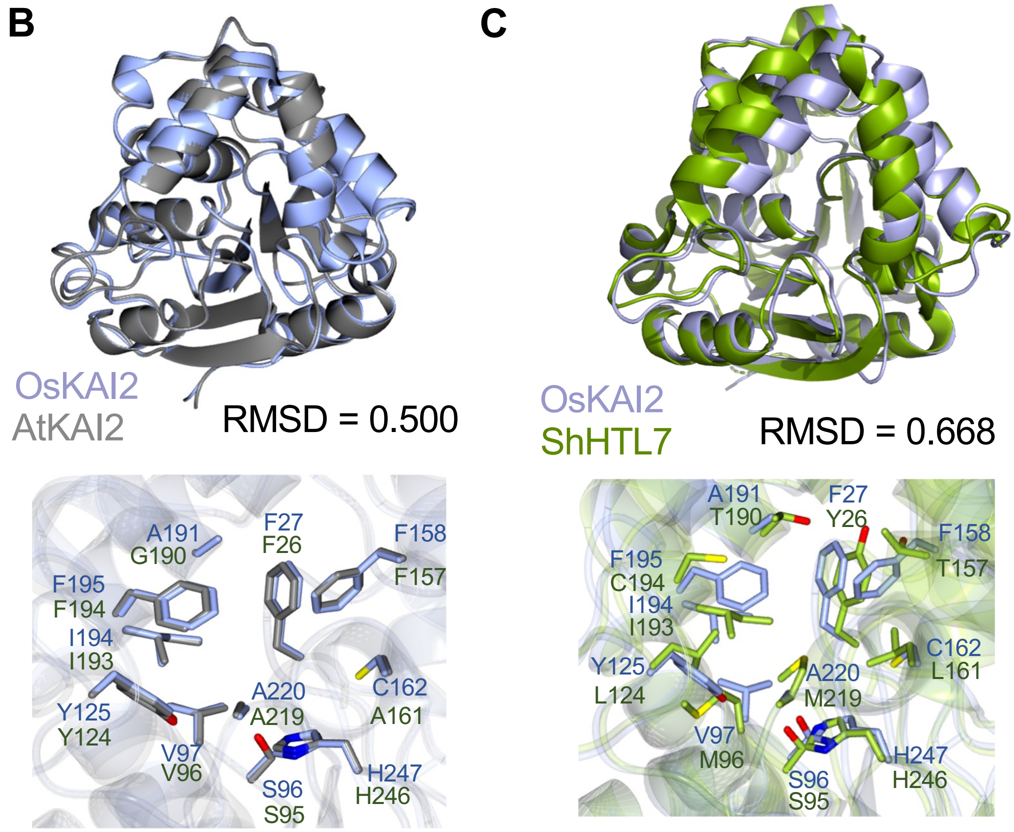

D

Protein	Surface area (A^2)	Volume (A^3)
OsKAI2	193.794	107.917
AtKAI2 (4HRY)	168.521	63.419
OsD14 (4IH9)	251.181	144.730
AtD14	253.963	148.290
ShHTL7 (5Z95)	299.684	215.173
ShD14 (5Z7Z)	283.260	153.069
PsKAI2 B (7K2Z)	234.425	125.377

Figure S2. Comparing overall structure and pocket residues of OsKAI2 with other KAI2/D14 proteins. (A) Binding pocket cavities in OsKAI2 (blue), AtKAI2 (grey), OsD14 (gold), AtD14 (orange), and ShHTL7 (green) are shown in grey surface. Superposition of OsKAI2 structure with **(B)** AtKAI2 and **(C)** ShHTL7 with corresponding RMSD for Cα. **(D)** Binding pocket dimensions are listed for a range of KAI2, D14, and HTL structures selected from the PDB as calculated by CASTp with a 1.4 Å radius probe.

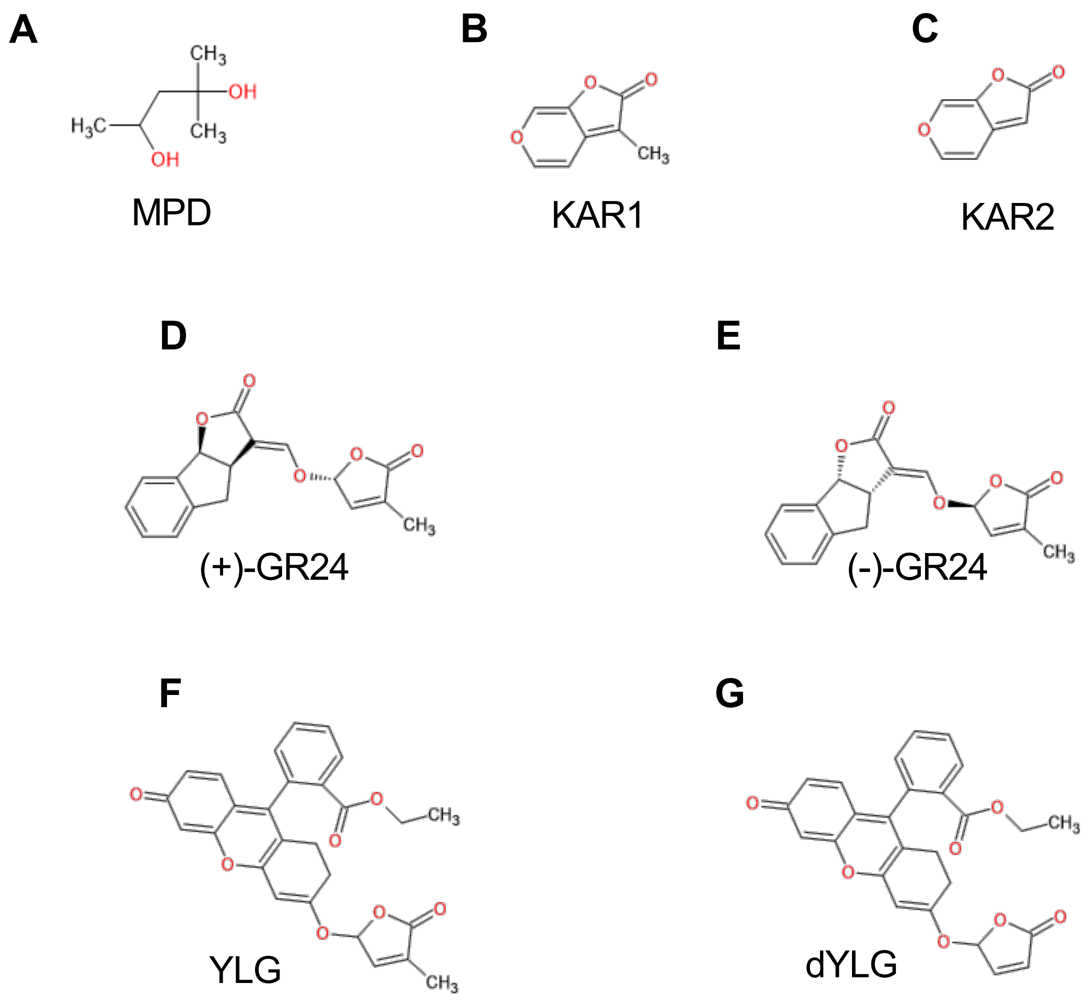

Figure S3. Structures of molecules discussed in this manuscript. (A) 2-Methyl-2,4-pentadiol (MPD) found in pocket of OsKAI2. Examples of Karrikin type molecules, KAR1 **(B)** and KAR2 **(C)**. A synthetic strigolactone (SL) ligand GR24 the (+) enantiomer **(D)** and (-) enantiomer **(E)**. **(F)** Yoshimulactone green (YLG), a synthetic pro-fluorescent strigolactone ligand typically used in enzyme assays. **(G)** Desmethyl yoshimulactone green (dYLG), a synthetic pro-fluorescent strigolactone ligand used in these studies.

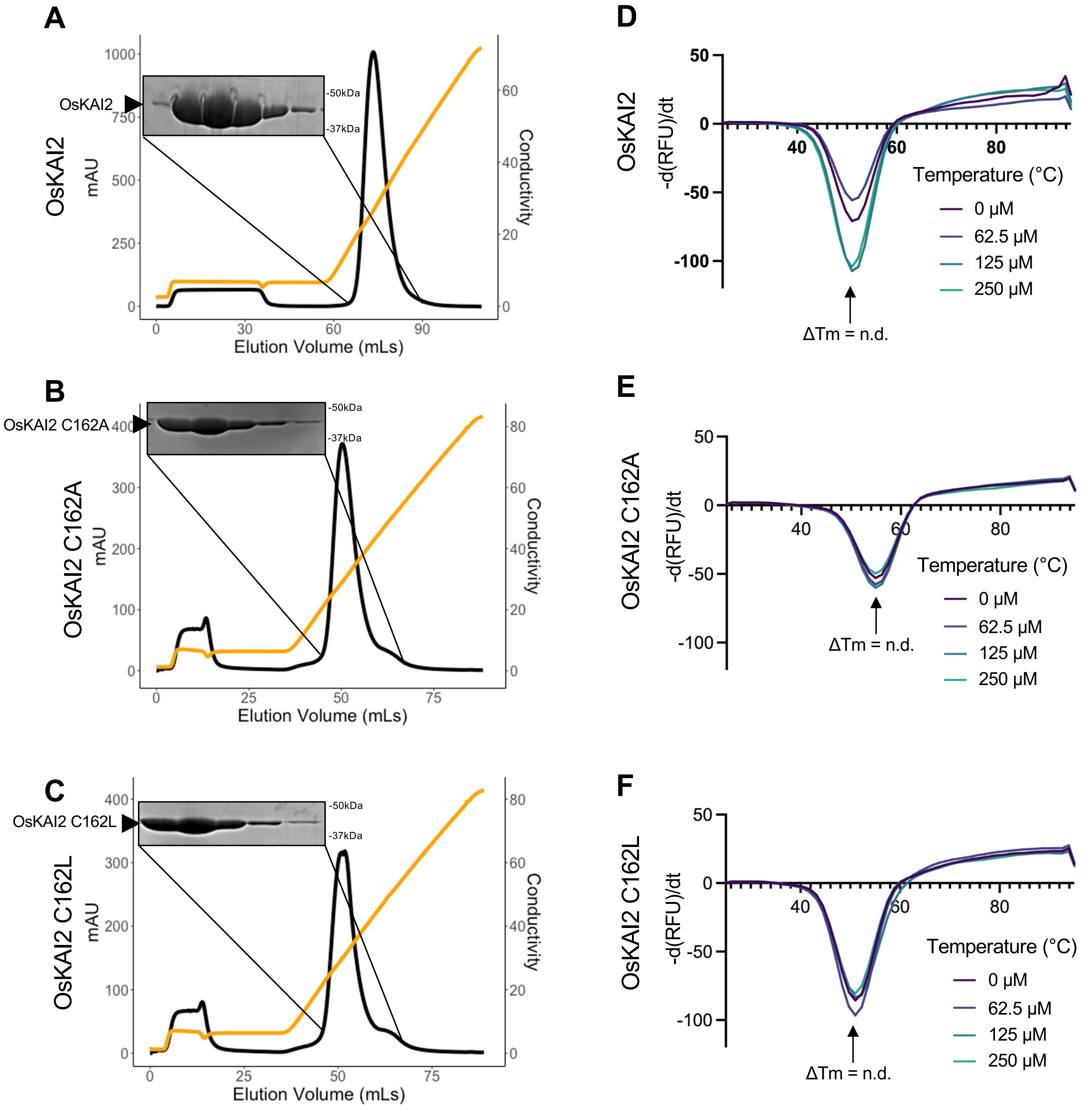

G

	k_{cat} (s^{-1})	K_m (μM)	K_i (μM)	k_{cat}/K_m ($s^{-1}μM^{-1}$)
WT	0.0263 ± 0.0111	16.4 ± 10.0	28.6 ± 18.6	0.00161 ± 0.00166
C162L	0.0162 ± 0.00432	15.0 ± 7.37	426 ± 645	0.00118 ± 0.000900
C162A	9.68 ± 8552	n.d.	0.0238 ± 21.0	n.d.

Figure S4. KAI2s used in this study and additional biochemical data. (A-C) Purification anion exchange step for KAI2s used in study. **(D-F)** DSF analysis of OsKAI2, OsKAI2 C162A and OsKAI2 C162L in response to (+)-GR24. (**G**) Kinetic parameters determined by initial rates kinetics assay using substrate inhibition fitting in Graphpad Prism.

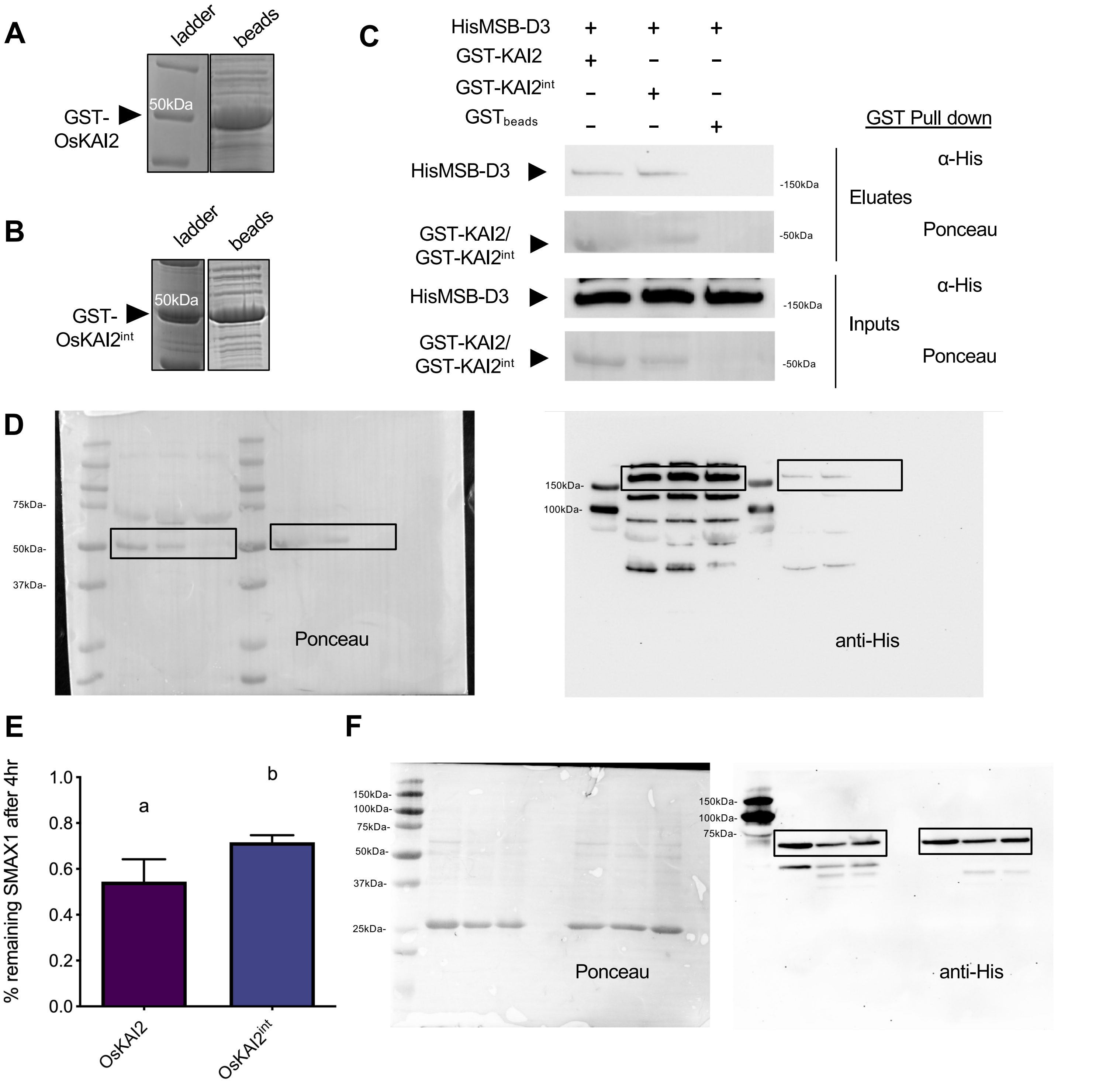

Figure S5. Reagents and uncropped gels corresponding to pull-down and in vitro degradation assays. GST-KAI2 **(A)** or GST-KAI2^{int} **(B)** -bound beads for use in pull-down assays. **(C)** Pull-down experiment of His-MSB tagged OsD3-ASK1 by GST-KAI2 or GST-KAI2^{int} mutant in the presence of (-)-GR24. Proteins were resolved by SDS-PAGE and were visualized via western blot analysis with ponceau and anti-His antibodies as indicated. **(D)** Uncropped gel of pull-down ponceau (left) and anti-His (right). Areas boxed represent areas cropped and used in panel **(C)**. **(E)** Mean values of three experimental replicas measuring HisMSB-OsSMAX1$_{D2}$ remaining levels relative to its T=0 in the presence of wildtype *Oryza sativa* total cell extract supplemented with recombinant KAI2 or KAI2int as indicated. Arbitrary units (au). (Student's t test $p < 0.05$). Proteins were resolved by SDS-PAGE and visualized via western blot with anti-His antibodies, protein bands were quantified in-silico. **(F)** Uncropped gel of in vitro degradation experiment ponceau (left) and anti-His (right). Areas boxed represent areas cropped and used in Figure 4 panel E.

	OsKAI2-‘apo’	OsKAI2-MPD	AtD14
**Data collection**			
Space group	$P1\ 2_1\ 1$	$P1\ 2_1\ 1$	$P1\ 2_1\ 1$
Cell dimensions			
a, b, c (Å)	37.64 85.39, 44.62	37.71, 86.48, 44.62	43.69, 69.27, 90.80
α, β, γ (°)	90.00, 114.91, 90.00	90.00, 114.94, 90.00	90.00 93.38 90.00
Resolution (Å)	42.73-1.68 (1.71-1.68)	40.49-1.29 (1.31-1.29)	45.32-2.00 (2.05-2.00)
R_{sym}	0.069 (0.422)	0.051 (0.320)	0.043 (0.317)
<I / σI>	16.0 (2.6)	19.7 (2.6)	14.6 (1.4)
Completeness (%)	93.8 (84.1)	99.3 (88.3)	99.3 (91.7)
CC1/2	0.99 (0.88)	0.99 (0.90)	0.99 (0.80)
Redundancy	3.5 (3.5)	4.3 (3.2)	5.8 (3.4)
**Refinement**			
Resolution (Å)	1.68	1.29	2.00
Unique reflections	27040 (1274)	64764 (2848)	36532 (2459)
R_{work} / R_{free} (%)	16.8/19.7	15.2/16.8	16.3/20.7
No. atoms	4158	4407	4357
Protein	4055	4138	4125
Ligand/ion	-	21	-
Water	103	245	232
B-factors			
Protein	22.7	14.7	33.85
Ligand/ion	-	15.1	-
Water	28.0	24.1	38.5
R.m.s. deviations			
Bond lengths (Å)	0.0084	0.0116	0.0067
Bond angles (°)	1.553	1.925	1.559
Ramachandran favored (%)	97.40	96.92	96.56
Ramachandran allowed (%)	2.60	3.08	3.06
Ramachandran outliers (%)	0	0	0.38
PDB ID	8VCZ	8VD1	8VD3

Table S1. Data collection, phasing and refinement statistics. *Statistics for the highest-resolution shell are shown in parentheses.

Residues/ B-factors	S96	H247	F195	F27	F158	I194	A191	V97	A220	Y125	C162
**OsKAI2-Apo**	15.37	17.65	33.17	22.28	23.49	18.93	19.21	15.59	17.88	21.38	22.05
**OsKAI2-MPD**	9.70	11.18	13.66	11.58	15.41	11.11	10.51	9.397	13.60	10.52	14.1
**AtD14**	30.48	31.63	35.88	24.28	54.82	37.66	35.35	27.83	56.82	31.9	37.97

Table S2. Comparison of B-factors for selected residues within the catalytic pocket. Table shown calculated average of residues within the catalytic pocket for the different crystal structures reported here.

		Species	Gene Name	Sequence ID	Source
MONOCOTs	Poaceae	Oryza sativa Japonica Group	probable esterase D14L	XP_015617518.1	NCBI
MONOCOTs	Poaceae	Panicum hallii	probable esterase D14L	XP_025798102.1	NCBI
MONOCOTs	Poaceae	Sorghum bicolor	probable esterase D14L	XP_002465088.1	NCBI
MONOCOTs	Poaceae	Lolium rigidum	probable esterase D14L	XP_047090814.1	NCBI
MONOCOTs	Poaceae	Brachypodium distachyon	probable esterase D14L	XP_003562367.1	NCBI
MONOCOTs	Poaceae	Triticum dicoccoides	probable esterase D14L	XP_037430390.1	NCBI
MONOCOTs	Poaceae	Aegilops tauschii subsp. strangulata	probable esterase D14L	XP_020178880.1	NCBI
MONOCOTs	Poaceae	Triticum urartu	probable esterase D14L	XP_048570933.1	NCBI
MONOCOTs	Poaceae	Hordeum vulgare subsp. vulgare	probable esterase D14L	XP_044981364.1	NCBI
MONOCOTs	Poaceae	Setaria italica	probable esterase D14L	XP_004983951.1	NCBI
MONOCOTs	Poaceae	Dichanthelium oligosanthes	putative esterase D14L	OEL18596.1	NCBI
MONOCOTs	Poaceae	Oryza brachyantha	probable esterase D14L	XP_006650237.1	NCBI
MONOCOTs	Poaceae	Panicum virgatum	probable esterase D14L	XP_039828974.1	NCBI
MONOCOTs	Poaceae	Mascanthus sinensis	ESTERASE KAI2-RELATED	v7.1|Misin01G312200.1.p	Phytozome
MONOCOTs	Poaceae	Mascanthus sinensis	ESTERASE KAI2-RELATED	v7.1|Misin02G301000.1.p	Phytozome
MONOCOTs	Poaceae	Chasmanthium laxum	ESTERASE KAI2-RELATED	v1.1|Chala.04G038000.1.p	Phytozome
MONOCOTs	Poaceae	Zea mays	ESTERASE KAI2-RELATED	RefGen_V4|Zm00001d029590_P001	Phytozome
MONOCOTs	Poaceae	Zea mays	ESTERASE KAI2-RELATED	RefGen_V4|Zm00001d047335_P001	Phytozome
MONOCOTs	Poaceae	Zea mays	ESTERASE KAI2-RELATED	RefGen_V4|Zm00001d029590_P001	Phytozome
MONOCOTs	Poaceae	Eleusine coracana	ESTERASE KAI2-RELATED	v1.1|ELECO.r07.3AG0229120.1	Phytozome
MONOCOTs	Poaceae	Eleusine coracana	ESTERASE KAI2-RELATED	v1.1|ELECO.r07.3BG0274580.1	Phytozome
MONOCOTs	Poaceae	Brachypodium sylvaticum	ESTERASE KAI2-RELATED	v2.1|Brasyl.2G222900.1.p	Phytozome
MONOCOTs	Poaceae	Brachypodium arbuscula	ESTERASE KAI2-RELATED	v3.1|Barbu.2G218500.1.p	Phytozome
MONOCOTs	Poaceae	Brachypodium hybridum	ESTERASE KAI2-RELATED	v2.1|Bhyb26.D01G152100.1.p	Phytozome
MONOCOTs	Poaceae	Brachypodium hybridum	ESTERASE KAI2-RELATED	v2.1|Bhyb26.S02G194500.1.p	Phytozome
MONOCOTs	Poaceae	Thinopyrum intermedium	ESTERASE KAI2-RELATED	v3.1|Thint.J04G190800.1.p	Phytozome
MONOCOTs	Poaceae	Thinopyrum intermedium	ESTERASE KAI2-RELATED	v3.1|Thint.S04G175500.1.p	Phytozome
MONOCOTs	Poaceae	Brachypodium mexicanum	ESTERASE KAI2-RELATED	v2.1|Bmexi.02PG369700.1.p	Phytozome
MONOCOTs	Poaceae	Brachypodium mexicanum	ESTERASE KAI2-RELATED	v2.1|Bmexi.02UG214100.1.p	Phytozome
MONOCOTs	Joinvilleaceae	Joinvillea ascendens	ESTERASE KAI2-RELATED	v1.1|Joasc.01G055100.1.p	Phytozome
MONOCOTs	Joinvilleaceae	Joinvillea ascendens	ESTERASE KAI2-RELATED	v1.1|Joasc.01G055000.1.p	Phytozome
MONOCOTs	Orchidaceae	Phalaenopsis equestris	probable esterase D14L	XP_020584281.1	NCBI
MONOCOTs	Orchidaceae	Apostasia shenzhenica	putative esterase KAI2	PKA58159.1	NCBI
MONOCOTs	Orchidaceae	Dendrobium catenatum	probable esterase D14L	XP_020704619.1	NCBI
MONOCOTs	Arecaceae	Elaeis guineensis	probable esterase D14L	XP_010905263.1	NCBI
MONOCOTs	Arecaceae	Phoenix dactylifera	probable esterase D14L	XP_008787141.3	NCBI
MONOCOTs	Cyperaceae	Carex littledalei	esterase D14L	KAF3323272.1	NCBI
MONOCOTs	Musaceae	Musa acuminata subsp. malaccensis	probable esterase D14L	XP_009421091.1	NCBI
MONOCOTs	Zingiberaceae	Zingiber officinale	probable esterase D14L	XP_042388420.1	NCBI
MONOCOTs	Bromeliaceae	Ananas comosus	probable esterase D14L	XP_020082399.1	NCBI
MONOCOTs	Dioscoreaceae	Dioscorea cayenensis subsp. rotundata]	probable esterase D14L	XP_039137355.1	NCBI
MONOCOTs	Dioscoreaceae	Dioscorea alata	ESTERASE KAI2-RELATED	v2.1|Dioal.13G030200.1.p	Phytozome
MONOCOTs	Asparagaceae	Asparagus officinalis	probable esterase D14L	XP_020242859.1	NCBI
MONOCOTs	Asparagaceae	Agave tequilana var. Weber's Blue	ESTERASE KAI2-RELATED	v2.1|AgateH1.08G198100.1.p	Phytozome
MONOCOTs	Acoraceae	Acorus americanus	ESTERASE KAI2-RELATED	v1.1|Acora.05G132600.1.p	Phytozome
MONOCOTs	Araceae	Spirodela polyrhiza	ESTERASE KAI2-RELATED	S.polyrhiza v2|Spipo2G0095700	Phytozome
BASAL ANGIOSPERMS and OTHER	Nymphaeaceae	Nymphaea colorata	probable esterase D14L	XP_031494429.1	NCBI
BASAL ANGIOSPERMS and OTHER	Amborellaceae	Amborella trichopoda	probable esterase D14L	XP_006829226.1	NCBI
BASAL ANGIOSPERMS and OTHER	Lauraceae	Cinnamomum micranthum f. kanehirae	putative esterase D14L	RWR81441.1	NCBI
BASAL ANGIOSPERMS and OTHER	Magnoliaceae	Liriodendron tulipifera	ESTERASE KAI2-RELATED	v1.1|Litul.17G055800.1.p	Phytozome
EUDICOTS	Proteaceae	Telopea speciosissima	probable esterase KAI2	XP_043715070.1	NCBI
EUDICOTS	Proteaceae	Macadamia integrifolia	probable esterase KAI2	XP_042493687.1	NCBI
EUDICOTS	Malvaceae	Gossypium hirsutum	probable esterase D14L	XP_016719056.1	NCBI
EUDICOTS	Malvaceae	Hibiscus syriacus	probable esterase KAI2	XP_038996839.1	NCBI
EUDICOTS	Malvaceae	Gossypium australe	probable esterase D14L	KAA3457435.1	NCBI
EUDICOTS	Malvaceae	Theobroma cacao	probable esterase D14L	XP_017969914.1	NCBI
EUDICOTS	Malvaceae	Herrania umbratica	probable esterase KAI2	XP_021274049.1	NCBI
EUDICOTS	Actinidiaceae	Actinidia eriantha	probable esterase D14L	XP_057514426.1	NCBI
EUDICOTS	Amaranthaceae	Chenopodium quinoa	probable esterase KAI2	XP_021714410.1	NCBI
EUDICOTS	Amaranthaceae	Beta vulgaris subsp. Vulgaris	probable esterase D14L	XP_010679577.1	NCBI
EUDICOTS	Amaranthaceae	Spinacia oleracea	ESTERASE KAI2-RELATED	S.oleracea Spov3|Spov3_chr3.01003	Phytozome
EUDICOTS	Amaranthaceae	Amaranthus hypochondriacus	ESTERASE KAI2-RELATED	v2.1|AH006186-RA	Phytozome
EUDICOTS	Celastraceae	Tripterygium wilfordii	probable esterase KAI2	XP_038723957.1	NCBI
EUDICOTS	Rhamnaceae	Ziziphus jujuba var. spinosa	probable esterase KAI2	XP_048335111.1	NCBI
EUDICOTS	Theaceae	Camellia lanceoleosa	probable esterase D14L	KAI8028962.1	NCBI
EUDICOTS	Myricaceae	Morella rubra	probable esterase D14L	KAB1214871.1	NCBI
EUDICOTS	Anacardiaceae	Pistacia vera	probable esterase KAI2	XP_031277949.1	NCBI
EUDICOTS	Caricaceae	Carica papaya	probable esterase KAI2	XP_021908336.1	NCBI
EUDICOTS	Papaveraceae	Papaver somniferum	probable esterase KAI2	XP_026394197.1	NCBI
EUDICOTS	Solanaceae	Solanum tuberosum	ESTERASE KAI2-RELATED	v6.1|Soltu.DM.02G007700.1	Phytozome
EUDICOTS	Solanaceae	Solanum tuberosum	ESTERASE KAI2-RELATED	v6.1|Soltu.DM.02G007690.1	Phytozome
EUDICOTS	Solanaceae	Solanum tuberosum	ESTERASE KAI2-RELATED	v6.1|Soltu.DM.02G028010.1	Phytozome
EUDICOTS	Brassicaceae	Arabidopsis thaliana	KAI2	AT4G37470.1	TAIR
EUDICOTS	Brassicaceae	Capsella rubella	ESTERASE KAI2-RELATED	v1.1|Carub.0007s0281.1.p	Phytozome

		Species	Gene Name	Sequence ID	Source
EUDICOTS	Phrymaceae	Erythranthe guttata	KAI2a	XP_012856373.1	NCBI
EUDICOTS	Phrymaceae	Erythranthe guttata	KAI2b	XP_012853813.1	NCBI
EUDICOTS	Phrymaceae	Erythranthe guttata	KAI2c	XP_012840794.1	NCBI
EUDICOTS	Vitaceae	Vitis vinifera	probable esterase KAI2	XP_002284043.1	NCBI
EUDICOTS	Salicaceae	Populus trichocarpa	KAI2a	XP_002307293.1	NCBI
EUDICOTS	Salicaceae	Populus trichocarpa	KAI2b	XP_006380654.1	NCBI
EUDICOTS	Rosaceae	Prunus persica	probable esterase KAI2	XP_007205717.1	NCBI
EUDICOTS	Fabaceae	Medicago truncatula	KAi2a	Mt4.0v1|Medtr4g095310.1	Phytozome
EUDICOTS	Fabaceae	Medicago truncatula	KAI2b	Mt4.0v1|Medtr5g016150.1	Phytozome
EUDICOTS	Fabaceae	Lotus Japonicus	KAI2a	Lj1.0v1|Lj2g0007403.1	Phytozome
EUDICOTS	Fabaceae	Lotus Japonicus	KAI2b	Lj1.0v1|Lj4g0019758.1	Phytozome
EUDICOTS	Fabaceae	Cicer arietinum	ESTERASE KAI2-RELATED	v1.0|Ca_02196	Phytozome
EUDICOTS	Fabaceae	Cicer arietinum	ESTERASE KAI2-RELATED	v1.0|Ca_09326	Phytozome
EUDICOTS	Fabaceae	Glycine max	ESTERASE KAI2-RELATED	v2.1|GmISU01.17G149900.1.p	Phytozome
EUDICOTS	Fabaceae	Glycine max	ESTERASE KAI2-RELATED	v2.1|GmISU01.17G150000.1.p	Phytozome
EUDICOTS	Fabaceae	Glycine max	ESTERASE KAI2-RELATED	v2.1|GmISU01.01G152200.1.p	Phytozome
EUDICOTS	Fabaceae	Glycine max	ESTERASE KAI2-RELATED	v2.1|GmISU01.11G045700.1.p	Phytozome
EUDICOTS	Fabaceae	Glycine max	ESTERASE KAI2-RELATED	v2.1|GmISU01.05G083300.1.p	Phytozome
EUDICOTS	Fabaceae	Pisum sativum	KAI2a		Guercio et al 2022
EUDICOTS	Fabaceae	Pisum sativum	KAI2b		Guercio et al 2022
EUDICOTS	Crassulaceae	Kalanchoe fedtschenkoi	ESTERASE KAI2-RELATED	v1.1|Kaladp0034s0001.1.p	Phytozome
EUDICOTS	Portulacaceae	Portulaca amilis	ESTERASE KAI2-RELATED	v1.0|FUN_030516-T1	Phytozome
EUDICOTS	Crassulaceae	Kalanchoe laxiflora	ESTERASE KAI2-RELATED	v3.1|Kalaxd.05G066100.1.p	Phytozome
EUDICOTS	Fagaceae	Castanea dentata	ESTERASE KAI2-RELATED	v1.1|Caden.02G181900.1.p	Phytozome
EUDICOTS	Ranunculaceae	Aquilegia coerulea	ESTERASE KAI2-RELATED	v3.1|Aqcoe6G293500.1.p	Phytozome
EUDICOTS	Ranunculaceae	Aquilegia coerulea	ESTERASE KAI2-RELATED	v3.1|Aqcoe6G293700.1.p	Phytozome
EUDICOTS	Ranunculaceae	Aquilegia coerulea	ESTERASE KAI2-RELATED	v3.1|Aqcoe6G293600.1.p	Phytozome
EUDICOTS	Ranunculaceae	Aquilegia coerulea	ESTERASE KAI2-RELATED	v3.1|Aqcoe1G368100.1.p	Phytozome
EUDICOTS	Hydrangeaceae	Hydrangea quercifolia	ESTERASE KAI2-RELATED	v1.1|Hyque.05G069100.1.p	Phytozome
EUDICOTS	Hydrangeaceae	Hydrangea quercifolia	ESTERASE KAI2-RELATED	v1.1|Hyque.05G069300.1.p	Phytozome
EUDICOTS	Asteraceae	Helianthus annuus	ESTERASE KAI2-RELATED	r1.2|HanXRQChr12g0373271	Phytozome
EUDICOTS	Asteraceae	Helianthus annuus	ESTERASE KAI2-RELATED	r1.2|HanXRQChr11g0322481	Phytozome
EUDICOTS	Asteraceae	Lactuca sativa	ESTERASE KAI2-RELATED	V8|Lsat_1_v5_gn_4_162120.1	Phytozome
EUDICOTS	Asteraceae	Lactuca sativa	ESTERASE KAI2-RELATED	V8|Lsat_1_v5_gn_4_162080.1	Phytozome
EUDICOTS	Oleaceae	Olea europaea	ESTERASE KAI2-RELATED	v1.0|Oeu011610.1	Phytozome
EUDICOTS	Oleaceae	Olea europaea	ESTERASE KAI2-RELATED	v1.0|Oeu040607.1	Phytozome
EUDICOTS	Apiaceae	Daucus carota	ESTERASE KAI2-RELATED	v2.0|DCAR_010837	Phytozome
EUDICOTS	Apiaceae	Daucus carota	ESTERASE KAI2-RELATED	v2.0|DCAR_017602	Phytozome
EUDICOTS	Rutaceae	Citrus sinensis	ESTERASE KAI2-RELATED	v1.1|orange1.1g046596m	Phytozome
EUDICOTS	Betulaceae	Betula platyphylla	ESTERASE KAI2-RELATED	v1.1|BPChr06G22594	Phytozome
EUDICOTS	Betulaceae	Betula platyphylla	ESTERASE KAI2-RELATED	v1.1|BPChr06G22550	Phytozome
EUDICOTS	Betulaceae	Betula platyphylla	ESTERASE KAI2-RELATED	B.platyphylla v1.1|BPChr06G22593	Phytozome
EUDICOTS	Orobanchaceae	Lindenbergia philippensis	ESTERASE KAI2-RELATED	v1.1|Liphi.09G063400.1.p	Phytozome
EUDICOTS	Orobanchaceae	Lindenbergia philippensis	ESTERASE KAI2-RELATED	v1.1|Liphi.04G117700.1.p	Phytozome
EUDICOTS	Orobanchaceae	Striga hermonthica	HTL1		Toh et al 2015
EUDICOTS	Orobanchaceae	Striga hermonthica	HTL2		Toh et al 2015
EUDICOTS	Orobanchaceae	Striga hermonthica	HTL3		Toh et al 2015
EUDICOTS	Orobanchaceae	Striga hermonthica	HTL4		Toh et al 2015
EUDICOTS	Orobanchaceae	Striga hermonthica	HTL5		Toh et al 2015
EUDICOTS	Orobanchaceae	Striga hermonthica	HTL6		Toh et al 2015
EUDICOTS	Orobanchaceae	Striga hermonthica	HTL7		Toh et al 2015
EUDICOTS	Orobanchaceae	Striga hermonthica	HTL8		Toh et al 2015
EUDICOTS	Orobanchaceae	Striga hermonthica	HTL9		Toh et al 2015
EUDICOTS	Orobanchaceae	Striga hermonthica	HTL10		Toh et al 2015
EUDICOTS	Orobanchaceae	Striga hermonthica	HTL11		Toh et al 2015
EUDICOTS	D14s	Arabidopsis thaliana	D14	AT3G03990.1	TAIR

Table S3. All sequences used in amino acid tree and dN/dS analysis.
